# Association between machine learning-assisted heavy metal exposures and diabetic kidney disease: a cross-sectional survey and Mendelian randomization analysis

**DOI:** 10.3389/fpubh.2024.1367061

**Published:** 2024-06-14

**Authors:** Ruiqi Zhao, Sen Lin, Mengyao Han, Zhimei Lin, Mengjiao Yu, Bei Zhang, Lanyue Ma, Danfei Li, Lisheng Peng

**Affiliations:** ^1^The Fourth Clinical Medical College of Guangzhou University of Chinese Medicine, Shenzhen, Guangdong, China; ^2^The Second Clinical Medical College of Guangzhou University of Chinese Medicine, Guangzhou, Guangdong, China; ^3^Shenzhen Traditional Chinese Medicine Hospital, Shenzhen, Guangdong, China

**Keywords:** environmental epidemiology, heavy metal exposures, diabetic kidney disease, NHANES, machine learning, Mendelian randomization

## Abstract

**Background and objective:**

Heavy metals, ubiquitous in the environment, pose a global public health concern. The correlation between these and diabetic kidney disease (DKD) remains unclear. Our objective was to explore the correlation between heavy metal exposures and the incidence of DKD.

**Methods:**

We analyzed data from the NHANES (2005–2020), using machine learning, and cross-sectional survey. Our study also involved a bidirectional two-sample Mendelian randomization (MR) analysis.

**Results:**

Machine learning reveals correlation coefficients of −0.5059 and − 0.6510 for urinary Ba and urinary Tl with DKD, respectively. Multifactorial logistic regression implicates urinary Ba, urinary Pb, blood Cd, and blood Pb as potential associates of DKD. When adjusted for all covariates, the odds ratios and 95% confidence intervals are 0.87 (0.78, 0.98) (*p* = 0.023), 0.70 (0.53, 0.92) (*p* = 0.012), 0.53 (0.34, 0.82) (*p* = 0.005), and 0.76 (0.64, 0.90) (*p* = 0.002) in order. Furthermore, multiplicative interactions between urinary Ba and urinary Sb, urinary Cd and urinary Co, urinary Cd and urinary Pb, and blood Cd and blood Hg might be present. Among the diabetic population, the OR of urinary Tl with DKD is a mere 0.10, with a 95%CI of (0.01, 0.74), urinary Co 0.73 (0.54, 0.98) in Model 3, and urinary Pb 0.72 (0.55, 0.95) in Model 2. Restricted Cubic Splines (RCS) indicate a linear linkage between blood Cd in the general population and urinary Co, urinary Pb, and urinary Tl with DKD among diabetics. An observable trend effect is present between urinary Pb and urinary Tl with DKD. MR analysis reveals odds ratios and 95% confidence intervals of 1.16 (1.03, 1.32) (*p* = 0.018) and 1.17 (1.00, 1.36) (*p* = 0.044) for blood Cd and blood Mn, respectively.

**Conclusion:**

In the general population, urinary Ba demonstrates a nonlinear inverse association with DKD, whereas in the diabetic population, urinary Tl displays a linear inverse relationship with DKD.

## Graphical abstract

IVW, inverse variance weighted; WM, weighted median.

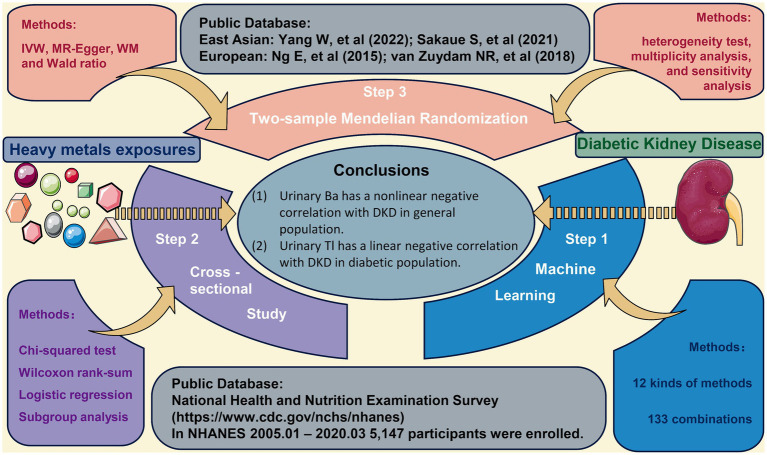

## 1 Introduction

Diabetes, a global health concern, affected roughly 537 million adults, about 10% of the world’s population, in 2021. It’s projected that by 2030, this figure will rise to 643 million and by 2045, it will reach 783 million (1). The kidneys are among the organs most impacted by diabetes, which is the leading cause of chronic kidney disease (CKD). Approximately 40% of people with diabetes develop diabetic kidney disease (DKD) (2, 3). Despite the effective therapeutic benefits of glucagon-like peptide-1 receptor agonist (GLP-1 RA) and sodium glucose cotransporter 2 (SGLT2) inhibitors in diabetes treatment, which also show substantial renal protective effects (4–6), CKD remains the primary cause of end-stage kidney disease (ESKD) in the United States (7).

Heavy metals, ubiquitously present in the environment, can permeate the human body via the skin, respiratory tract, and digestive system (8–10). This indicates a potential threat of heavy metal exposures to the general populace through sources such as potable water, food, and dermal contact. Environmental exposure to heavy metals has emerged as a global public health concern (11). Previous research has explored the correlation between heavy metal exposures and diabetes (12–17), and numerous studies have also examined the link between heavy metal exposures and renal functionality (18–22). A cross-sectional study revealed a correlation between Cd and Pb exposure and DKD (23). Furthermore, an animal experiment showed that after exposure to Cd, compared with normal rats, diabetic rats had a more obvious increase in urinary microalbumin/creatinine, and the expression of autophagy related proteins, apoptosis and fibrosis proteins were higher. Cd exposure may via inhibiting autophagy aggravates diabetic kidney damage (24). Nonetheless, a unified agreement remains elusive. Up to the present, a limited number of scholars have delved into the potential link between heavy metal exposure and DKD, leaving the existence of a correlation between heavy metal exposure and DKD as an open question.

Therefore, we embarked on a systematic evaluation of the correlation between heavy metal exposures and the incidence of DKD, utilizing data from the National Health and Nutrition Examination Survey (NHANES) (2005.01–2020.03) in the United States. To corroborate the association between heavy metal exposures and DKD, and to investigate their potential causative link, we employed a combination of traditional cross-sectional survey methods, novel machine learning approaches, and Mendelian randomization (MR) studies. Traditional logistic regression and subgroup analyses are commonly applied to cross-sectional surveys. In recent years, epidemiologists have increasingly applied machine learning methods in model building. Machine learning demonstrates higher efficiency and lower error in analyzing complex relationships between variables and handling data extremes, while also exhibiting flexibility and robustness (25–27). MR is a method that uses genetic variants as instrumental variables (IVs) to effectively avoid various biases that can arise in observational clinical studies, balancing confounding factors, and is commonly used to infer causal relationships between exposures and outcomes (28). The comprehensive procedure of this study is depicted in Graphical Abstract.

## 2 Materials and methods

### 2.1 The study population in NHANES

Conducted by the National Center for Health Statistics (NCHS) in the United States, the NHANES is a cross-sectional study employing a stratified, multistage probability sampling method to representatively sample the U.S. population. The survey spans a broad spectrum of areas, encompassing interviews, physical examinations, dietary assessments, and laboratory tests, among other components (29). In the current study, we scrutinized data from seven cycles, ranging from January 2005 to March 2020. The research protocol for this period received approval from the Institutional Review Board of the NCHS, and written informed consent was obtained from all participants. Within the span from January 2005 to March 2020, a total of 76,496 individuals participated in the NHANES survey. Our study encompassed 43,412 participants aged 20 years and above, excluding 740 pregnant women. From this cohort, we further included 5,150 subjects with complete records for crucial variables, including urinary and blood heavy metal levels, age, gender, race, poverty status, education level, and biochemical indicators. We excluded one participant who declined to disclose their education level and two participants with ambiguous smoking status. Ultimately, a total of 5,147 participants were incorporated into our study. The meticulous selection process is illustrated in Supplementary Figure S1.

### 2.2 Heavy metal exposures and other covariates used in NHANES

Utilizing the data sourced from NHANES, our study incorporated measurements of nine urinary and three blood heavy metal concentrations, obtained across seven cycles from January 2005 to March 2020, for the evaluation of heavy metal exposure. The urinary heavy metals investigated encompassed barium (Ba), cadmium (Cd), cobalt (Co), cesium (Cs), molybdenum (Mo), lead (Pb), antimony (Sb), thallium (Tl), and tungsten (W). Conversely, the blood heavy metals comprised cadmium (Cd), lead (Pb), and mercury (Hg).

In addition to the main variables, we integrated participants’ age, gender, race, education level, income status, smoking habits, physical activity, body mass index, presence of hypertension, hyperlipidemia, hyperuricemia, normal liver function, UACR, and eGFR as covariates to mitigate confounding influences. In this investigation, a poverty index below 2 was used to classify participants as low income individuals. Educational attainment was bifurcated into two categories: high school or below and above high school. Body weight status was gauged using three BMI thresholds: 18.5, 25, and 30, to categorize participants as Underweight, Normal, Overweight, or Obese. Abnormal liver function was determined using cutoffs of ALT ≥43 U/L (for males) or GGT > 58 U/L (for males), and ALT ≥31 U/L (for females) or GGT > 35 U/L (for females). Hypertensive status was assigned to participants previously diagnosed with hypertension or those currently on hypertension medications. Hyperlipidemia was defined as having total cholesterol ≥240 mg/dL, triglycerides ≥200 mg/dL, LDL-C ≥ 160 mg/dL, HDL-C ≤ 40 mg/dL, or a prior diagnosis of hyperlipidemia or use of related medications (30). Hyperuricemia is defined as a blood uric acid ≥6.0 mmol/L (31). Participants were classified as engaging in moderate-to-vigorous physical activity if they self-reported undertaking either moderate or vigorous occupational or recreational activities. In terms of smoking status, participants were categorized as follows: “never smokers” (those who have smoked fewer than 100 cigarettes in their lifetime), “former smokers” (individuals who have previously smoked 100 or more cigarettes but have since ceased), and “current smokers” (those who have smoked 100 or more cigarettes and continue to do so). Comprehensive details regarding the questionnaire surveys, measurement methodologies, and instruments employed can be sourced from the corresponding sections within each respective study cycle (29).

### 2.3 Definition and assessment of DKD in NHANES

The diagnostic criteria for diabetes include meeting at least one of the following conditions: (1) fasting plasma glucose ≥7.0 mmol/L. (2) glycosylated hemoglobin (HbA1c) ≥ 6.5 mmol/L. (3) prior medical diagnosis of diabetes by a physician or healthcare expert. (4) current use of diabetes medication or insulin. Among individuals with diabetes, participants who meet at least one of the following criteria are defined as having DKD: (1) UACR ≥30 mg/g. (2) eGFR <60 mL/min/1.73m^2^. (3) Diabetes affected eyes/had retinopathy (32). The eGFR was calculated using the 4-variable Modification of Diet in Renal Disease (MDRD-4) equation (33). The diagnostic criteria for diabetes and DKD were based on the relevant guidelines provided by the American Diabetes Association (ADA) (32, 34).

### 2.4 GWAS data and single nucleotide polymorphisms selection

We performed MR analysis on East Asian and European cohorts to control for potential confounding by population characteristics. The Asian GWAS data, which pertained to heavy metal exposures, were sourced from a study involving 2,488 Chinese participants. Specifically, this data comprised the serum and plasma levels of 16 heavy metals: barium (Ba), lead (Pb), cobalt (Co), molybdenum (Mo), cadmium (Cd), vanadium (V), chromium (Cr), aluminum (Al), manganese (Mn), nickel (Ni), tin (Sn), titanium (Ti), rubidium (Rb), strontium (Sr), copper (Cu), and zinc (Zn) (35). The East Asian GWAS data for DKD were collected from a Japanese study (36). For the European cohort, we utilized GWAS data from a Swedish study of 949 participants, which included whole blood levels of 11 heavy metals (Al, Cd, Co, Cu, Cr, Hg, Mn, Mo, Ni, Pb, and Zn) (37). The European GWAS data for DKD were extracted from a composite dataset (38). All referenced studies were ethically approved, and informed consent was obtained from all participants. Detailed characteristics of these GWAS studies can be found in the respective publications, with a summary provided in Supplementary Table S1.

Firstly, a genome-wide significance threshold of *p* < 5 × 10–5 was employed to identify genetic variants strongly associated with the exposure. Additionally, SNPs with missing key information were excluded from the analysis. In addition, SNPs in substantial linkage disequilibrium (defined as a distance of 10,000 kb, linkage disequilibrium *r*^2^ < 0.001) were excluded. Subsequently, the *F*-value for each SNPs was computed using the formula *F* = BETA^2^exposure/SE^2^exposure (39), and SNPs with *F* < 10 were further removed. Moreover, palindrome SNPs were also eliminated. Detailed information on the SNPs included in the MR analysis can be found in Supplementary Table S2.

### 2.5 Statistical analysis

We executed a tripartite statistical investigation to probe the plausible correlation between heavy metal exposures and DKD.

In the first phase, the baseline data of 5,147 participants were compared based on whether they had DKD. We employed descriptive statistics, such as the median, interquartile range (IQR), and percentages. Initial evaluations were carried out using the chi-squared test with Rao and Scott’s second-order correction, as well as the Wilcoxon rank-sum test for complex survey samples. We applied machine learning to NHANES data on nine urinary heavy metals levels and DKD status. Initially, we randomly segregated the 5,147 participants into a validation group and a control group via random sampling. Within a cross-validation framework, we utilized one algorithm for variable selection, while harnessing another for the construction of the predictive classification model. The R packages used for machine learning analysis are as follows: randomForestSRC, glmnet, plsRglm, gbm, caret, mboost, e1071, BART, MASS, snowfall, xgboost. The area under the curve (AUC) was calculated for all 113 model combinations used in the dataset, which included both the training and validation sets. Ultimately, we selected the best model based on its AUC for risk model construction. The risk score was computed as follows:


$$Risk\;Score=\sum_{i=1}^{n}\;Coe\int_{i}\;x_{i}$$

Where $Coe\underset{i}{\int }$ represents the risk coefficient and $x_{i}$ symbolizes the urinary heavy metal variables.

In the second phase, we established three multivariate logistic regression models for verification. In Model 1, adjustments were made solely for age (continuous). In Model 2, we accounted for age (continuous), gender, race, educational attainment, income stratum, smoking status, physical activity, and BMI. In Model 3, hypertension, hyperlipidemia, hyperuricemia, liver enzyme levels, UACR, and eGFR were further incorporated into the multifactorial regression model. Additionally, we probed the interactions between heavy metal exposures within Model 3. To bolster the credibility of our conclusions, subgroup analyses were conducted within the diabetic population, and three models were similarly fitted. We also employed Restricted Cubic Spline (RCS) to discern whether the relationship between exposure and outcome is linear or nonlinear. If the nonlinear association proved insignificant, urinary and blood heavy metal concentrations were converted into discrete variables, based on the quartiles of the weighted sample distribution, to further conduct trend analysis. At this stage, given the multi-stage complex sampling survey’s characteristics, all analyses incorporated the survey weights of each participant.

During the final phase, we separately performed bidirectional two-sample MR analyses in East Asian and European populations to explore any causal or reverse causal relationships between heavy metal exposures and DKD. The TwoSampleMR software package was utilized for these bidirectional two-sample MR analysis, which incorporated the Inverse variance weighting (IVW), Weighted median estimator (WM), MR-Egger, and Wald ratio for MR analysis. We conducted heterogeneity tests using IVW and MR-Egger, and implemented outlier detection as required using MR-PRESSO. Further, the intercept of MR-Egger was employed to evaluate horizontal pleiotropy. Lastly, a Leave-one-out sensitivity test was executed to examine the potential influence of individual SNPs on the MR results. Supplementary Figure S2 provides a detailed research flowchart for this phase.

We executed the analyses for the three previously mentioned stages utilizing R software (Version 4.2.2). For all analyses, we established statistical significance as a two-sided *p*-value <0.05.

## 3 Results

### 3.1 Demographic characteristics

Participant demographic details and baseline comparisons are elaborated in Table 1. We enrolled a total of 5,147 participants, median age being 47 years, with a balanced dispersion across all age brackets. The cohort comprised 2,558 males and 2,589 females, with Non-Hispanic Whites forming the majority. Among these, 416 individuals were diagnosed with DKD. Predominantly, DKD patients were aged above 60 years and frequently possessed an educational background not surpassing high school. When juxtaposed with non-DKD participants, a larger fraction of high-income individuals, former smokers, and those engaging in less physical activity were observed among DKD patients. Furthermore, obesity, hypertension, hyperlipidemia, elevated UACR, diminished eGFR, and a higher prevalence of increased liver enzymes were frequently found among DKD participants. Nonetheless, the fraction of participants with hyperuricemia was comparatively lower.

**Table 1:** Baseline characteristics of participants categorized by participants without and with DKD.

Variable	Overall *N* = 5147^a^	DKD		*p-*value^b^
		Without	With	
		*N* = 4731^a^	*N* = 416^a^	
**Age**	47 (34, 60)	46 (33, 59)	65 (54, 73)	<0.001**
**Age (group)**				<0.001**
*20–40 years*	1,776 (38%)	1,750 (39%)	26 (11%)	
*40–60 years*	1,744 (38%)	1,636 (39%)	108 (28%)	
*60+ years*	1,627 (24%)	1,345 (22%)	282 (61%)	
**Gender**				0.14
*Female*	2,589 (50%)	2,403 (51%)	186 (45%)	
*Male*	2,558 (50%)	2,328 (49%)	230 (55%)	
**Race**				0.2
*Mexican American*	777 (8.3%)	700 (8.2%)	77 (11%)	
*Other Hispanic*	503 (5.5%)	470 (5.6%)	33 (4.0%)	
*Non-Hispanic White*	2,218 (69%)	2,047 (69%)	171 (66%)	
*Non-Hispanic Black*	1,060 (10%)	963 (10%)	97 (13%)	
*Other Race – Including Multi-Racial*	589 (7.1%)	551 (7.2%)	38 (6.6%)	
**Education level**				<0.001**
*High school or below*	2,406 (39%)	2,146 (38%)	260 (59%)	
*High school above*	2,741 (61%)	2,585 (62%)	156 (41%)	
**Income level**				<0.001**
*Low income*	2,694 (67%)	2,528 (67%)	166 (52%)	
*High income*	2,453 (33%)	2,203 (33%)	250 (48%)	
**Smoker status**				0.037*
*Never smoker*	2,818 (54%)	2,601 (54%)	217 (52%)	
*Former smoker*	1,294 (27%)	1,149 (26%)	145 (34%)	
*Current smoker*	1,035 (19%)	981 (20%)	54 (13%)	
**Physical activity**				<0.001**
*Moderate work activity below*	1,428 (22%)	1,253 (21%)	175 (35%)	
*Moderate work activity or above*	3,719 (78%)	3,478 (79%)	241 (65%)	
**BMI**				<0.001**
*Underweight*	1,443 (29%)	1,384 (30%)	59 (11%)	
*Normal*	91 (1.4%)	89 (1.4%)	2 (0.6%)	
*Overweight*	1,698 (33%)	1,582 (33%)	116 (28%)	
*Obese*	1,915 (37%)	1,676 (35%)	239 (60%)	
**Hypertension**				<0.001**
*No*	3,330 (69%)	3,197 (71%)	133 (38%)	
*Yes*	1,817 (31%)	1,534 (29%)	283 (62%)	
**Hyperlipidemia**				<0.001**
*No*	2,395 (48%)	2,297 (49%)	98 (28%)	
*Yes*	2,752 (52%)	2,434 (51%)	318 (72%)	
**Hyperuricemia**				<0.001**
*No*	3,378 (67%)	3,180 (68%)	198 (50%)	
*Yes*	1,769 (33%)	1,551 (32%)	218 (50%)	
**Liver enzyme**				0.002**
*Normal*	4,168 (82%)	3,870 (83%)	298 (72%)	
*High*	979 (18%)	861 (17%)	118 (28%)	
**UACR (mg/g)**	6 (4, 11)	6 (4, 10)	38 (11, 110)	<0.001**
**eGFR (mL/min/1.73 m** ^**2**^ **)**	87 (74, 102)	88 (76, 102)	67 (53, 93)	<0.001**
**Metals, urine**				
*Barium (Ba), urine (ug/L)*	1.19 (0.59, 2.19)	1.23 (0.60, 2.22)	0.76 (0.40, 1.60)	<0.001**
*Cadmium (Cd), urine (ug/L)*	0.25 (0.13, 0.45)	0.24 (0.13, 0.44)	0.29 (0.16, 0.53)	<0.001**
*Cobalt (Co), urine (ug/L)*	0.33 (0.20, 0.53)	0.34 (0.20, 0.53)	0.32 (0.20, 0.50)	0.5
*Cesium (Cs), urine (ug/L)*	4.6 (2.8, 6.9)	4.6 (2.8, 6.9)	4.3 (3.0, 6.8)	0.6
*Molybdenum (Mo), urine (ug/L)*	37 (21, 63)	37 (21, 63)	40 (24, 63)	0.13
*Lead (Pb), urine (ug/L)*	0.39 (0.21, 0.67)	0.39 (0.21, 0.68)	0.39 (0.24, 0.63)	0.5
*Antimony (Sb), urine (ug/L)*	0.05 (0.03, 0.08)	0.05 (0.03, 0.08)	0.05 (0.03, 0.08)	0.5
*Thallium (Tl), urine (ug/L)*	0.17 (0.10, 0.26)	0.17 (0.10, 0.27)	0.14 (0.09, 0.21)	<0.001**
*Tungsten (W), urine (ug/L)*	0.06 (0.03, 0.11)	0.06 (0.03, 0.11)	0.07 (0.04, 0.12)	0.064
**Metals, blood**				
*Cadmium (Cd), blood (ug/L)*	0.29 (0.18, 0.52)	0.29 (0.18, 0.52)	0.35 (0.20, 0.53)	0.2
*Lead (Pb), blood (ug/dL)*	1.06 (0.70, 1.70)	1.05 (0.69, 1.69)	1.22 (0.84, 1.76)	0.010**
*Mercury (Hg), blood (ug/L)*	0.85 (0.43, 1.66)	0.85 (0.43, 1.66)	0.71 (0.39, 1.60)	0.11

a Median (IQR) for continuous variables; *n* (%) for categorical variables.

b Chi-squared test with Rao & Scott's second-order correction for categorical variables; Wilcoxon rank-sum test for complex survey samples for continuous variables.

DKD, diabetic kidney disease; UACR, urinary albumin-to-creatinine ratio; eGFR, estimated glomerular filtration rate. *p-value < 0.05; **p-value < 0.01. The bold font indicates *p*-value < 0.05.

### 3.2 Associations of urinary metals with DKD in machine learning

**Figure 1:**
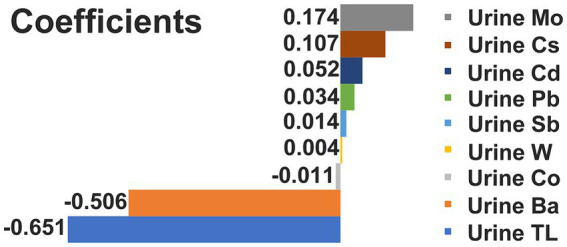
Correlation coefficient between urinary heavy metal exposures and DKD.

We devised 113 risk prediction models utilizing methodologies such as Lasso, Ridge, Enet, Stepglm, Support Vector Machine (SVM), glmBoost, Linear Discriminant Analysis (LDA), plsRglm, RandomForest, Gradient Boosting Machine (GBM), XGBoost, and NaiveBayes, and portrayed their AUC (Supplementary Figure S3). Evident from the figure, the RandomForest (RF) model exhibited commendable predictive prowess in both training and validation sets, boasting AUC values of 1.000 and 0.715, correspondingly. Based on this, we graphically represented urinary heavy metal concentrations highly associated with DKD as determined by RF (Figure 1). The figure clearly illustrates that urinary Ba (coefficient = −0.5059) and urinary Tl (coefficient = −0.6510) bear an inverse correlation with the incidence of DKD, while the correlation coefficients between other urinary heavy metals and DKD are negligible.

### 3.3 Associations of heavy metal exposures with DKD in multivariate logistics regression

**Figure 2:**
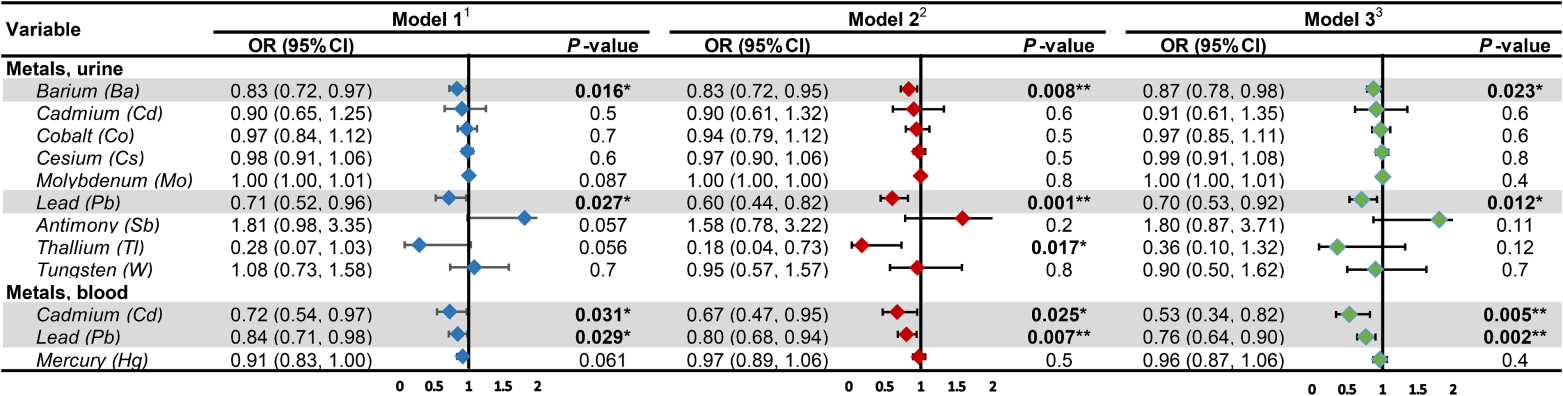
Associations of heavy metal exposures with diabetic kidney disease in general population. ^1^Model 1: age (continuous) was adjusted. ^2^Model 2: age (continuous), gender, race, education level, income level, smoker status, physical activity, BMI were adjusted. ^3^Model 3: age (continuous), gender, race, education level, income level, smoker status, physical activity, BMI, hypertension, hyperlipidemia, hyperuricemia, liver enzyme, UACR, eGFR were adjusted. UACR, urinary albumin-to-creatinine ratio; eGFR, estimated glomerular filtration rate; OR, odds ratio; CI, confidence interval. *p-value <0.05; **p-value <0.01.

**Figure 3:**
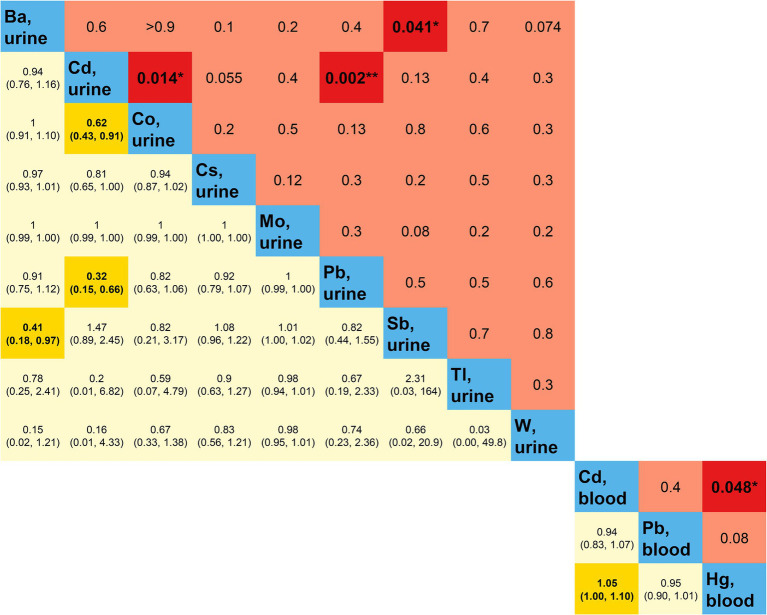
Multiplicative interactions between heavy metal exposures. Blue represents heavy metal exposure; yellow (below blue) represents the OR (95% CI) of the multiplicative interaction between two heavy metal exposures; red (above blue) represents the *p*-value of the multiplicative interaction between two heavy metal exposures. Darker colors indicate statistical significance. OR, odds ratio; CI, confidence interval. *p-value <0.05; **p-value <0.01.

**Figure 4:**
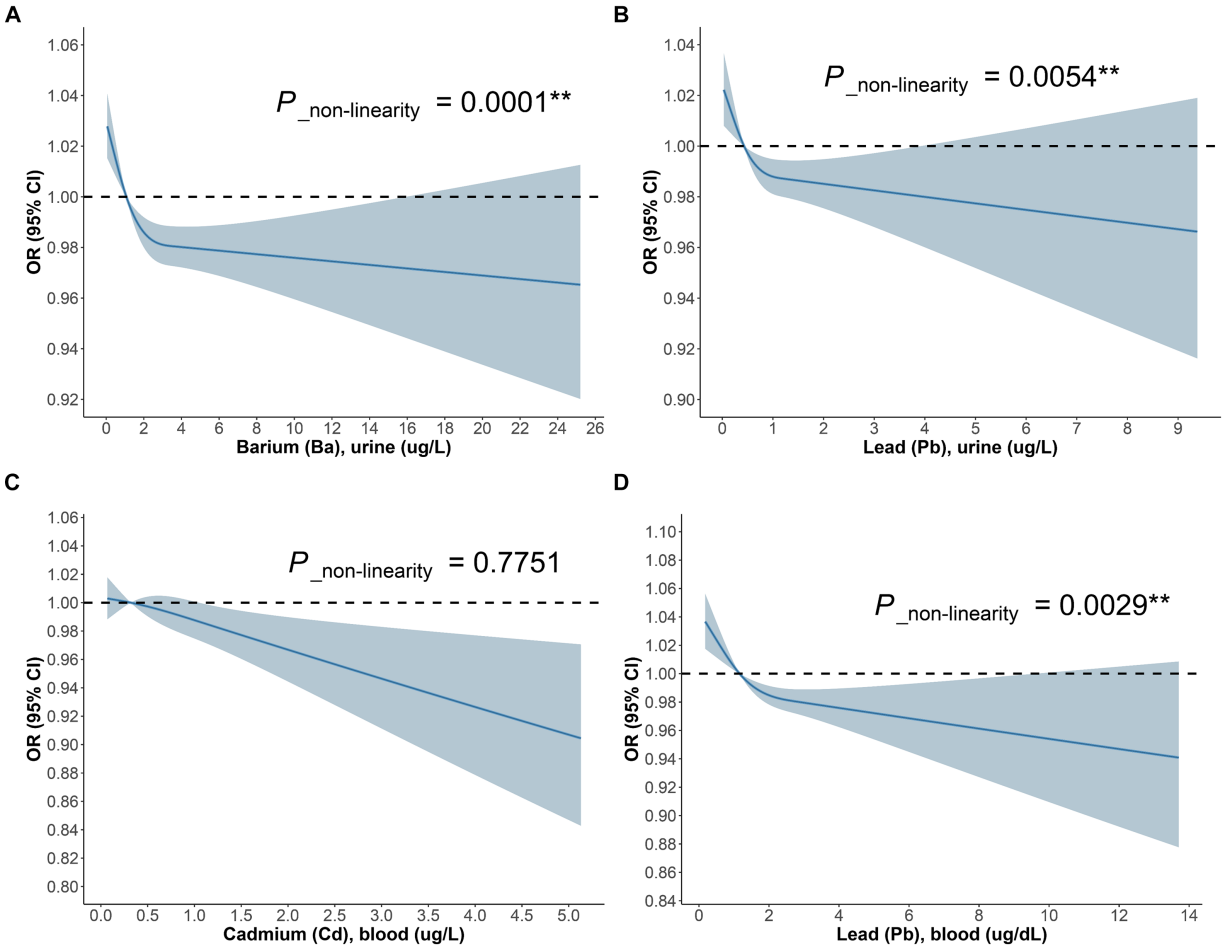
The dose–response relationship between **(A)** Ba urine, **(B)** Pb urine, **(C)** Cd blood, **(D)** Pb blood and DKD in general population.

The outcomes of the multifactorial logistic regression (Figure 2) demonstrate that urinary Ba, urinary Pb, blood Cd, and blood Pb consistently exhibit significant associations with DKD across all three models. In Model 3, accounting for all covariates, their odds ratios (ORs) (95% CIs) and *p* values are 0.87 (0.78, 0.98), *p* = 0.023; 0.70 (0.53, 0.92), *p* = 0.012; 0.53 (0.34, 0.82), *p* = 0.005; and 0.76 (0.64, 0.90), *p* = 0.002, correspondingly. For each unit increase in urinary Ba, urinary Pb, blood Cd, and blood Pb, the risk of DKD decreases by 13, 30, 47, and 24%, respectively. Furthermore, we scrutinized potential interactions between heavy metal levels in urine and blood (Figure 3). We identified potential interactions between urinary Ba and urinary Sb, with an OR = 0.41, 95% CI of (0.18, 0.97), and *p* = 0.041. Noteworthy interactions were discerned between urinary Cd and urinary Co, with an OR = 0.62, 95% CI of (0.43, 0.91), and *p* = 0.014. Significant interactions were found between urinary Cd and urinary Pb, with an OR = 0.32, 95% CI of (0.15, 0.66), and *p* = 0.002. Potential interactions might exist between blood Cd and blood Hg, with an OR = 1.05, 95% CI of (1.00, 1.10), and *p* = 0.048. We subsequently employed RCS to plot the dose–response relationship curves between these four heavy metals and DKD (Figure 4). We discerned that the correlation between blood Cd and the prevalence of DKD is linear (*P*_non-linearity = 0.7751), while the associations between urinary Ba (*P*_non-linearity = 0.0001), urinary Pb (*P*_non-linearity = 0.0054), and blood Pb (*P*_non-linearity = 0.0029) with DKD are nonlinear.

### 3.4 Associations of heavy metal exposures with DKD in subgroup analysis

**Figure 5:**
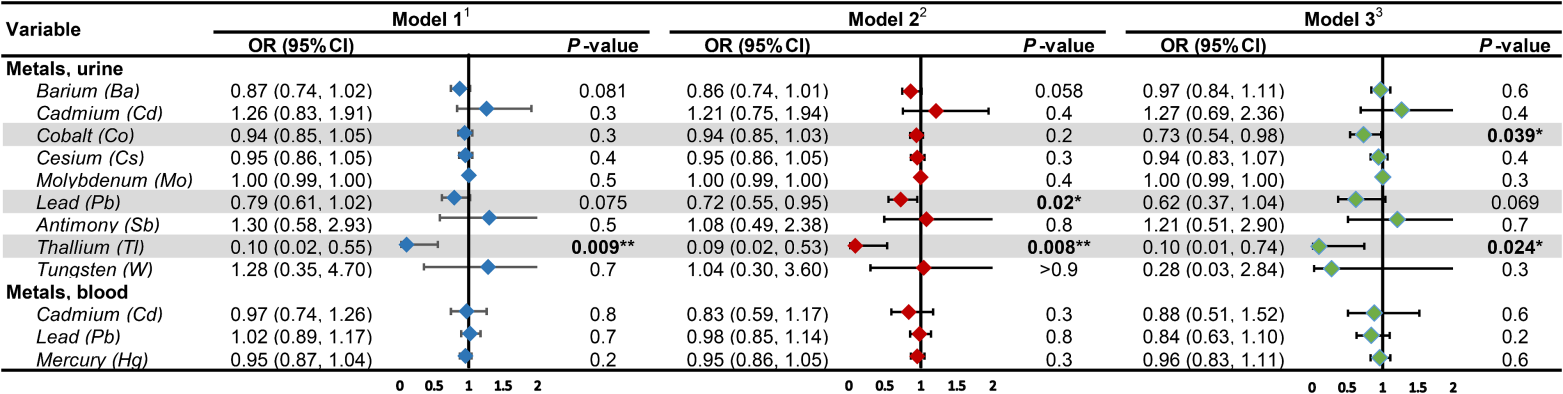
Associations of heavy metal exposures with diabetic kidney disease in diabetic population. ^1^Model 1: age (continuous) was adjusted. ^2^Model 2: age (continuous), gender, race, education level, income level, smoker status, physical activity, BMI were adjusted. ^3^Model 3: age (continuous), gender, race, education level, income level, smoker status, physical activity, BMI, hypertension, hyperlipidemia, hyperuricemia, liver enzyme, UACR, eGFR were adjusted. UACR, urinary albumin-to-creatinine ratio; eGFR, estimated glomerular filtration rate; OR, odds ratio; CI, confidence interval. *p-value <0.05; **p-value <0.01.

**Figure 6:**
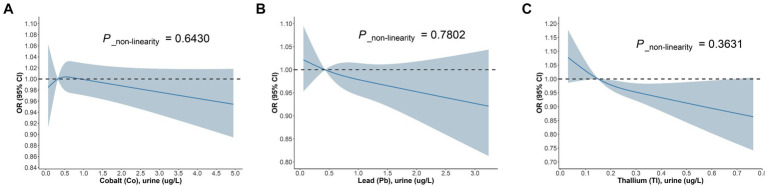
The dose–response relationship between **(A)** Co urine, **(B)** Pb urine, **(C)** Tl urine and DKD in diabetic population.

Likewise, we devised three models within the diabetic cohort (Figure 5). The study discovered that among diabetic patients, the correlation between urinary Co and DKD was significant solely in Model 3, with an OR = 0.73, 95% CI of (0.78, 0.98), and *p* = 0.039. For each unit increase in urinary Co, the risk of DKD in diabetic patients reduces to 73% of the initial. The association between urinary Pb and DKD was significant merely in Model 2, with an OR = 0.72, 95% CI of (0.55, 0.95), and *p* = 0.02. For each unit increase in urinary Pb, the prevalence of DKD dwindles by 28%. Besides, in the diabetic cohort, the correlation between urinary Tl and DKD was significant across all three models, with ORs (95% CIs) and *p* values being 0.1 (0.02, 0.55), *p* = 0.009; 0.09 (0.02, 0.53), *p* = 0.008; and 0.1 (0.01, 0.74), *p* = 0.024, respectively. In Model 3, for each unit increase in urinary Tl, the risk of DKD in diabetic patients diminishes to merely 10% of the original. Concurrently, the RCS model exhibits (Figure 6) that in the diabetic population, urinary Co, urinary Pb, and urinary Tl all bear a linear correlation with the prevalence of DKD (*P*_non-linearity are 0.6430, 0.7802, and 0.3631, respectively).

### 3.5 Associations of heavy metal exposures with DKD in trend analysis

**Figure 7:**
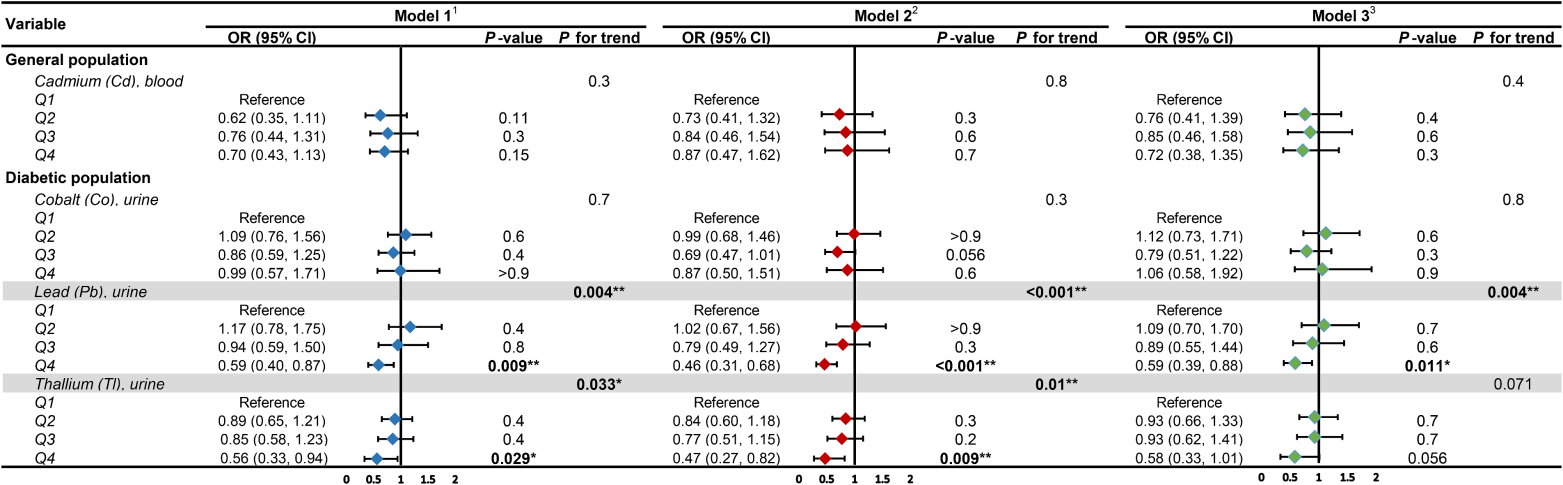
Forest plot for trend analysis. ^1^Model 1: age (continuous) was adjusted. ^2^Model 2: age (continuous), gender, race, education level, income level, smoker status, physical activity, BMI were adjusted. ^3^Model 3: age (continuous), gender, race, education level, income level, smoker status, physical activity, BMI, hypertension, hyperlipidemia, hyperuricemia, liver enzyme, UACR, eGFR were adjusted. UACR, urinary albumin-to-creatinine ratio; eGFR, estimated glomerular filtration rate; OR, odds ratio; CI, confidence interval. *p-value <0.05; **p-value <0.01.

We converted heavy metal exposure into discrete variables (Q1, Q2, Q3, Q4) based on quartiles and fitted the same three models to examine the heavy metal exposures that were significantly linearly related to DKD (Figure 7). The results revealed that within the diabetic population, with the lowest quartile (Q1) as the reference, the urinary Pb concentration in the highest quartile (Q4) consistently demonstrated significant correlation with DKD across all three models. The ORs (95% CIs) and *p* values of the three models were 0.59 (0.40, 0.87), *p* = 0.009; 0.46 (0.31, 0.68), *p* < 0.001; and 0.59 (0.39, 0.88), *p* = 0.011, respectively. The linear trend was consistently significant, with *P* for trend all lower than 0.05, indicating a significant linear inverse correlation between urinary Pb concentration and DKD in the diabetic population. Similarly, the linear trend of urinary Tl concentration with DKD in the diabetic population was only significant in Model 1 and Model 2 (*P* for trend both less than 0.05).

### 3.6 Associations of heavy metal exposures with DKD in MR analysis

**Figure 8:**
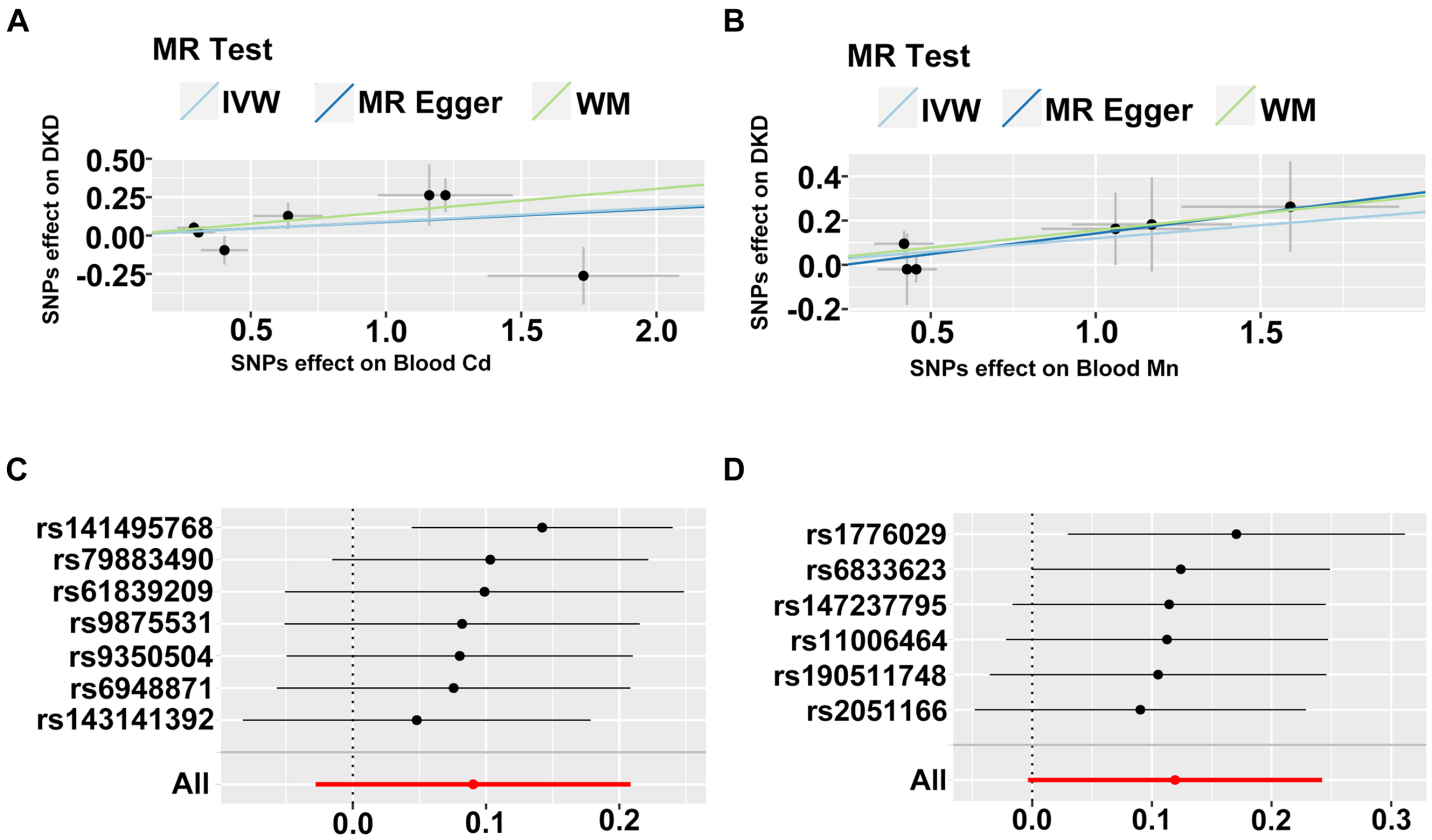
**(A,B)** The scatter plot of two sample mendelian randomization analysis. **(C,D)** The forest map based on the analysis result of “leave-one-out method”.

Finally, we executed a bidirectional two-sample MR analysis to further probe the causal relationship between heavy metal exposures and DKD. Where the final count of included SNPs was 2, solely the IVW method was employed for MR analysis. When the number of SNPs was 1, only the Wald ratio method was utilized for MR analysis. In other instances, we applied IVW, MR-Egger, and WM methods for MR analysis. We conducted bidirectional two-sample MR analysis in both East Asian and European populations. Due to database limitations, when DKD was used as exposure for MR analysis in the European population, we could not locate suitable SNPs to serve as instrumental variables. All MR analysis results are displayed in Supplementary Table S3. We identified potential causal relationships only between blood Cd and blood Mn and DKD in the European population. We subsequently visualized the MR outcomes of the two (Figures 8A,B). From the figure, it is discernible that blood Cd (WM: OR = 1.16, 95%CI is (1.03, 1.32), *p* = 0.018) and blood Mn (WM: OR = 1.17, 95%CI is (1.00, 1.36), *p* = 0.044) bear a positive correlation with DKD. We then applied IVW and MR-Egger methods to test for heterogeneity. The IVW (Q = 10.614, *p* = 0.101) and MR-Egger (Q = 10.608, *p* = 0.060) between blood Cd and DKD, and the IVW (Q = 2.666, *p* = 0.751) and MR-Egger (Q = 2.371, *p* = 0.668) between blood Mn and DKD all generated *p* values greater than 0.05, indicating no heterogeneity. We also calculated the intercept of MR-Egger to examine horizontal pleiotropy. The intercept between blood Cd and DKD is 0.003, *p* = 0.960; the intercept between blood Mn and DKD is −0.045, *p* = 0.615. Both intercepts approach 0, and *P* is greater than 0.05, indicating no horizontal pleiotropy. Lastly, we conducted a Leave-one-out sensitivity test (Figures 8C,D). The results reveal that upon removal of a certain SNP, the remaining SNPs can obtain results similar to the overall outcomes when performing MR analysis, thus no single SNP exerts a significant influence on the results.

## 4 Discussion

Based on surveys, limited studies have ventured into exploring the association between heavy metal exposures and DKD. In this study, we initially and systematically evaluated the correlation between heavy metal exposures and DKD. Machine learning unveiled a relationship between urinary Ba and urinary Tl with DKD, with correlation coefficients more pronounced than other urinary heavy metals. Findings from the cross-sectional study suggest that in the general population, urinary Ba and urinary Pb exhibit a significant negative correlation with the prevalence of DKD, as do blood Cd and blood Pb. In the diabetic cohort, urinary Tl bears a negative correlation with the prevalence of DKD, while urinary Co and urinary Pb only demonstrate a significant negative correlation with DKD in certain models. On the flip side, through MR analysis, we discovered a positive correlation between blood Cd, blood Mn, and DKD in the European population.

### 4.1 Barium

Our research indicates that in the general population, there exists a negative correlation between urinary Ba and DKD, and the RCS suggests a nonlinear relationship between the two. However, a similar conclusion was not reached within the diabetic population. Moreover, although the MR analysis in both East Asian and European populations did not find a causal link between serum, plasma, and whole blood Ba and DKD, this does not contradict the findings derived from this cross-sectional study. This is not only due to the source of the test samples and differences among the surveyed populations, but also because the MR analysis initially presumes a linear relationship between exposure and outcome, while the cross-sectional study suggests a nonlinear relationship between the two. To date, no researchers have investigated the association between Ba exposure and DKD, and several studies have demonstrated that Ba exposure is a risk factor for diabetes and renal function decline (13, 19, 40–42). Research has demonstrated that several metal elements, including Ba, may be linked with a higher eGFR in early pregnancy (43). An early animal experiment suggested that blood sugar levels diminished in rats exposed to Ba (44). Interestingly, a cross-sectional study found no difference in serum Ba levels between non-diabetic individuals and those with diabetes, but the concentration of Ba in the tears of non-diabetic individuals was lower than those with diabetes (45). To conclude, the correlation between Ba exposure and DKD remains undetermined. According to our investigation, our study may be the first to report on the association between Ba exposure and DKD.

### 4.2 Lead

Our study results indicate that in the general population, the levels of urinary Pb and blood Pb consistently demonstrate a nonlinear negative correlation with DKD. In the diabetic population, this negative correlation is also nonlinear and exhibits a significant trend effect. When Pb enters the human body through pathways such as the respiratory or gastrointestinal tract, it typically accumulates in the blood, soft tissues, and bones (46, 47). The half-life of Pb in these three locations is 35 days (in blood) (48), 40 days (in soft tissues) (49), and 20–30 years (in bones) (50), respectively. Furthermore, although Pb is primarily excreted from the body through urine, the amount of Pb excreted is quite low. Moreover, the Pb content in adult bones accounts for 80–95% of the total, suggesting that the Pb levels in blood and urine cannot accurately reflect chronic Pb exposure (46, 47, 51, 52). Although previous studies have demonstrated that higher Pb exposure is often associated with kidney damage (53–56), as mentioned earlier, blood Pb cannot serve as a qualified indicator for assessing Pb exposure. Moreover, we cannot discount reverse causality, leaving the “chicken or egg” question unanswered.

### 4.3 Cadmium

Cd is virtually omnipresent in all foods, making it impossible for humans to avoid exposure to Cd in daily life. Cd can infiltrate various cells, including renal tubular epithelial cells (57–59) and pancreatic β cells (60). The half-life of Cd in the blood ranges from approximately 75 to 128 days (61), and the average lifespan of red blood cells is 120 days, thus blood Cd concentration can serve as an indicator of recent exposure. However, on the flip side, due to the extremely low excretion and the human body’s inability to eliminate Cd, most of the Cd in the human body accumulates in the kidneys. An animal experiment indicated that Cd can only be excreted when cells die (62), therefore, using the Cd content in blood or urine as an evaluation indicator of long-term Cd exposure has certain limitations. In this study, we only observed a negative correlation between blood Cd and the prevalence of DKD in the general population. Interestingly, the MR analysis results in the European population showed that blood Cd is a risk factor for DKD. The reason for this result may be attributed to the significant differences in the participants of the two studies. The participants of the MR study selected from the GWAS data of the European population are the older adult (37), while the cross-sectional study involves the general population, and the overall blood Cd level is significantly lower than that of the older adult. Some studies suggest that Cd exposure may promote the progression of DKD in diabetic patients (63, 64). Overall, our MR research results support this conclusion.

### 4.4 Thallium

Tl is a recognized toxic heavy metal with good water solubility. It is mainly absorbed through the skin and mucous membranes, and vegetables are the main source of human Tl exposure (65). Thallium poisoning can affect various organs in the human body. Its biological half-life is 3–8 days and is primarily excreted through urine (66). Currently, there is no evidence of a correlation between low-dose Tl exposure and DKD. We found that in the diabetic population, urinary Tl and DKD are linearly negatively correlated, and there is a significant trend effect. Whether there exists a causal relationship between Tl exposure and DKD remains unknown. However, due to the high toxicity of Tl metal and its better water solubility compared to other metals, we cannot help but speculate that the decrease in urinary Tl content is due to the decline in renal filtration function in DKD.

### 4.5 Other heavy metal exposures

In addition to the aforementioned heavy metal exposures, we also discovered that urinary Co in the diabetic population demonstrated a significant linear negative correlation with DKD in Model 3, which adjusted for all covariates. Furthermore, in the MR analysis, we found that blood Mn might be a risk factor for DKD in the European population. However, these pieces of evidence only suggest potential correlations between heavy metal exposure and DKD, but they do not provide definitive conclusions. On the other hand, in this study, we did not find evidence of a correlation between other heavy metal exposures and DKD.

### 4.6 Strengths and limitations

In summary, we utilized data from the NHANES survey conducted from 2005 to March 2020, enabling a comprehensive investigation of the correlation between heavy metal exposure and DKD using a large sample size. The rigorous data collection procedures of NHANES and the professionally trained staff ensured the reliability of our research. Moreover, we combined novel machine learning statistical methods with traditional chi-square tests, rank-sum tests, and logistic regression analyses to validate our research results from multiple perspectives. We considered sampling weights in our analysis, making our conclusions more robust and, to some extent, generalizable to the adult population in the United States. Furthermore, we conducted subgroup analyses, evaluated the association between heavy metal exposure and DKD in the diabetic population, incorporated RCS curves into our analysis models, and conducted interaction and trend analyses. To compensate for the inability of cross-sectional studies to determine causality, we conducted bidirectional two-sample MR analyses in East Asian and European populations to assess the causal relationship between heavy metal exposures and DKD.

Despite our preliminary and systematic evaluation of the correlation between heavy metal exposures and DKD, this study has some limitations. Firstly, considering the differences in the half-life of heavy metals in the human body, the concentrations of heavy metals in urine and blood may not necessarily serve as qualified indicators for evaluating heavy metal exposures. Moreover, the metabolism of urine and blood may affect the measurement results. Secondly, we were unable to obtain the heavy metal content in other body tissues (such as hair, nails, bones, or organs) to assess heavy metal exposure. As the distribution of different heavy metals in the body varies, measuring the heavy metal content in urine and blood only once as a variable for assessing heavy metal exposures prevents us from accurately estimating the degree of heavy metal accumulation in the body. Thirdly, we did not use oral glucose tolerance test (OGTT) as a diagnostic criterion for diabetes, which may have resulted in fewer diagnosed diabetic and DKD patients than in reality. Then, due to database limitations, we did not include other heavy metal exposures or harmful substance exposures, so we cannot rule out these confounding factors. Finally, in the bidirectional two-sample MR analysis, we were unable to obtain more extensive GWAS data on heavy metal exposure. The GWAS data on heavy metal exposures included in this study had a small sample size and were limited to specific populations. We were unable to conduct reverse MR analysis in the European population, nor could we generalize the results of the MR to the general population.

## 5 Conclusion

In conclusion, our study provides evidence for the correlation between certain heavy metals and DKD, particularly urinary Ba in the general population and urinary Tl in the diabetic population, correlations that have not been reported previously. Our research findings may hold certain value for public environmental health. Considering the limitations of cross-sectional studies and two-sample MR studies, more rigorous prospective cohort studies and mechanistic studies are needed in the future to validate our conclusions.

## Data availability statement

Publicly available datasets were analyzed in this study. This data can be found at: https://wwwn.cdc.gov/Nchs/Nhanes/, https://www.nature.com/articles/s42003-022-03351-7, https://www.nature.com/articles/s41588-021-00931-x, https://academic.oup.com/hmg/article/24/16/4739/745575, https://diabetesjournals.org/diabetes/article/67/7/1414/35345/A-Genome-Wide-Association-Study-of-Diabetic-Kidney.

## Ethics statement

Ethical approval was not required for the studies involving humans because the data used in this study are publicly available. Therefore, no additional ethical approval or informed consent is necessary for secondary analysis. The studies were conducted in accordance with the local legislation and institutional requirements. The human samples used in this study were acquired from the data used in this study are publicly available. Written informed consent to participate in this study was not required from the participants or the participants’ legal guardians/next of kin in accordance with the national legislation and the institutional requirements.

## Author contributions

RZ: Conceptualization, Data curation, Formal analysis, Investigation, Methodology, Resources, Software, Supervision, Visualization, Writing – original draft, Writing – review & editing. SL: Conceptualization, Data curation, Formal analysis, Investigation, Methodology, Resources, Software, Supervision, Visualization, Writing – original draft, Writing – review & editing. MH: Conceptualization, Data curation, Formal analysis, Investigation, Methodology, Resources, Software, Supervision, Visualization, Writing – original draft, Writing – review & editing. ZL: Investigation, Validation, Writing – review & editing. MY: Resources, Validation, Writing – review & editing. BZ: Data curation, Formal analysis, Writing – review & editing. LM: Validation, Writing – review & editing. DL: Validation, Writing – review & editing. LP: Conceptualization, Funding acquisition, Methodology, Project administration, Supervision, Writing – original draft, Writing – review & editing.
