# Supplementary material for: Association between machine learning-assisted heavy metal exposures and diabetic kidney disease: a cross-sectional survey and Mendelian randomization analysis
Source: Front Public Health. 2024 Jun 14;12:1367061. doi: 10.3389/fpubh.2024.1367061 (PMC11212833; doi:10.3389/fpubh.2024.1367061)
Supplement: Supplementary file 1 [file Data_Sheet_1.docx]

Supplementary Material

# Supplementary Tables

| **Table S1.** Characteristics of GWAS enrolled in the MR study. | | | | | | | |
| --- | --- | --- | --- | --- | --- | --- | --- |
| **Exposure** | **Numbers in**  **GWAS Catalog** | **Sample size** | **Number of strongly**  **related SNPs** | **Number of finally enrolled SNPs** | | ***P*-value** | **Population** |
| **Serum barium levels** | GCST90100519 | 1,792 | 14 | | 5 | <5e-06 | East Asian |
| **Serum lead levels** | GCST90100530 | 1,765 | 24 | | 7 | <5e-06 | East Asian |
| **Serum cobalt levels** | GCST90100522 | 1,758 | 8 | | 2 | <5e-06 | East Asian |
| **Serum molybdenum levels** | GCST90100528 | 1,752 | 101 | | 5 | <5e-06 | East Asian |
| **Serum cadmium levels** | GCST90100521 | 1,775 | 12 | | 5 | <5e-06 | East Asian |
| **Serum vanadium levels** | GCST90100536 | 1,800 | 54 | | 7 | <5e-06 | East Asian |
| **Serum chromium levels** | GCST90100523 | 1,758 | 95 | | 5 | <5e-06 | East Asian |
| **Serum aluminum levels** | GCST90100517 | 1,775 | 20 | | 3 | <5e-06 | East Asian |
| **Serum manganese levels** | GCST90100527 | 1,674 | 19 | | 2 | <5e-06 | East Asian |
| **Serum nickel levels** | GCST90100529 | 1,756 | 154 | | 8 | <5e-06 | East Asian |
| **Serum tin levels** | GCST90100533 | 1,793 | 7 | | 1 | <5e-06 | East Asian |
| **Serum titanium levels** | GCST90100535 | 1,796 | 54 | | 5 | <5e-06 | East Asian |
| **Serum rubidium levels** | GCST90100531 | 1,795 | 14 | | 4 | <5e-06 | East Asian |
| **Serum strontium levels** | GCST90100534 | 1,797 | 100 | | 8 | <5e-06 | East Asian |
| **Serum copper levels** | GCST90100524 | 1,798 | 53 | | 6 | <5e-06 | East Asian |
| **Serum zinc levels** | GCST90100537 | 1,798 | 62 | | 5 | <5e-06 | East Asian |
| **Plasma barium levels** | GCST90100540 | 687 | 18 | | 8 | <5e-06 | East Asian |
| **Plasma lead levels** | GCST90100551 | 676 | 13 | | 2 | <5e-06 | East Asian |
| **Plasma cobalt levels** | GCST90100543 | 639 | 18 | | 3 | <5e-06 | East Asian |
| **Plasma molybdenum levels** | GCST90100549 | 682 | 5 | | 2 | <5e-06 | East Asian |
| **Plasma cadmium levels** | GCST90100542 | 656 | 15 | | 4 | <5e-06 | East Asian |
| **Plasma vanadium levels** | GCST90100557 | 688 | 13 | | 2 | <5e-06 | East Asian |
| **Plasma chromium levels** | GCST90100544 | 685 | 35 | | 3 | <5e-06 | East Asian |
| **Plasma aluminum levels** | GCST90100538 | 678 | 3 | | 2 | <5e-06 | East Asian |
| **Plasma manganese levels** | GCST90100548 | 681 | 6 | | 1 | <5e-06 | East Asian |
| **Plasma nickel levels** | GCST90100550 | 685 | 33 | | 5 | <5e-06 | East Asian |
| **Plasma tin levels** | GCST90100554 | 667 | 1 | | 1 | <5e-06 | East Asian |
| **Plasma titanium levels** | GCST90100556 | 688 | 14 | | 3 | <5e-06 | East Asian |
| **Plasma rubidium levels** | GCST90100552 | 688 | 3 | | 2 | <5e-06 | East Asian |
| **Plasma strontium levels** | GCST90100555 | 687 | 3 | | 2 | <5e-06 | East Asian |
| **Plasma copper levels** | GCST90100545 | 685 | 62 | | 3 | <5e-06 | East Asian |
| **Plasma zinc levels** | GCST90100558 | 684 | 5 | | 3 | <5e-06 | East Asian |
| **Blood aluminum levels** | NA | 949 | 7 | | 4 | <5e-06 | European |
| **Blood cadmium levels** | NA | 949 | 15 | | 7 | <5e-06 | European |
| **Blood cobalt levels** | NA | 949 | 12 | | 3 | <5e-06 | European |
| **Blood chromium levels** | NA | 949 | 11 | | 3 | <5e-06 | European |
| **Blood copper levels** | NA | 949 | 27 | | 9 | <5e-06 | European |
| **Blood mercury levels** | NA | 949 | 12 | | 3 | <5e-06 | European |
| **Blood manganese levels** | NA | 949 | 16 | | 6 | <5e-06 | European |
| **Blood molybdenum levels** | NA | 949 | 9 | | 4 | <5e-06 | European |
| **Blood nickel levels** | NA | 949 | 14 | | 8 | <5e-06 | European |
| **Blood lead levels** | NA | 949 | 15 | | 3 | <5e-06 | European |
| **Blood zinc levels** | NA | 949 | 12 | | 4 | <5e-06 | European |
| **Diabetic kidney disease** | GCST90018612 | 132,984 | 30 | | 4 in serum metal levels 3 in plasma metal levels | <5e-06 | East Asian |
| **Diabetic kidney disease** | GCST005881 | 10,875 | 59 | | 0 | <5e-06 | European |
| MR, mendelian randomization; GWAS, genome-wide association studies; SNPs, single nucleotide polymorphisms; *P*-value, the significance level of SNPs. | | | | | | | |

**Table S2.** The information for finally enrolled SNPs.

| **Exposure** | **Outcome** | **SNPs** | **EA-E** | **NEA-E** | **Beta-E** | **SE-E** | ***P*-E** | ***F*** | **EA-O** | **NEA-O** | **Beta-O** | **SE-O** | ***P*-O** | **Population** |
| --- | --- | --- | --- | --- | --- | --- | --- | --- | --- | --- | --- | --- | --- | --- |
| Serum Ba | DKD | rs145368539 | A | G | 0.4058 | 0.0886 | 5E-06 | 20.99 | A | G | -0.6279 | 0.2986 | 0.0355 | East Asian |
| Serum Ba | DKD | rs58616750 | T | C | -0.1672 | 0.0361 | 4E-06 | 21.44 | T | C | 0.048 | 0.0967 | 0.6199 | East Asian |
| Serum Ba | DKD | rs61857946 | C | T | 0.3446 | 0.0734 | 3E-06 | 22.06 | C | T | 0.1775 | 0.1497 | 0.2358 | East Asian |
| Serum Ba | DKD | rs7358335 | T | C | -0.1937 | 0.0413 | 3E-06 | 22.03 | T | C | 0.0665 | 0.1151 | 0.5633 | East Asian |
| Serum Ba | DKD | rs9364230 | T | C | -0.155 | 0.0332 | 3E-06 | 21.82 | T | C | -0.0243 | 0.0964 | 0.8007 | East Asian |
| Serum Pb | DKD | rs10991100 | A | C | -0.3014 | 0.0653 | 4E-06 | 21.33 | A | C | 0.0828 | 0.2037 | 0.6844 | East Asian |
| Serum Pb | DKD | rs11639885 | A | G | -0.297 | 0.0589 | 5E-07 | 25.43 | A | G | -0.0891 | 0.1289 | 0.4892 | East Asian |
| Serum Pb | DKD | rs12101120 | C | T | -0.1548 | 0.0338 | 5E-06 | 20.97 | C | T | -0.1358 | 0.0969 | 0.1612 | East Asian |
| Serum Pb | DKD | rs12617786 | A | G | 0.1604 | 0.0345 | 4E-06 | 21.66 | A | G | -0.1462 | 0.0959 | 0.1275 | East Asian |
| Serum Pb | DKD | rs2887641 | T | C | 0.172 | 0.0353 | 1E-06 | 23.69 | T | C | 0.0259 | 0.104 | 0.8035 | East Asian |
| Serum Pb | DKD | rs45576138 | A | G | 0.3998 | 0.0841 | 2E-06 | 22.58 | A | G | -0.0512 | 0.2039 | 0.8016 | East Asian |
| Serum Pb | DKD | rs79481489 | G | A | 0.4446 | 0.095 | 3E-06 | 21.88 | G | A | 0.3105 | 0.2279 | 0.1732 | East Asian |
| Serum Co | DKD | rs10255372 | G | A | -0.1583 | 0.0333 | 2E-06 | 22.59 | G | A | -0.041 | 0.0973 | 0.6738 | East Asian |
| Serum Co | DKD | rs991173 | G | A | -0.1681 | 0.0365 | 4E-06 | 21.22 | G | A | 0.1808 | 0.1018 | 0.0757 | East Asian |
| Serum Mo | DKD | rs111260116 | C | A | 0.1831 | 0.0381 | 2E-06 | 23.05 | C | A | 0.1299 | 0.0982 | 0.186 | East Asian |
| Serum Mo | DKD | rs12693546 | T | C | 0.3943 | 0.0807 | 1E-06 | 23.89 | T | C | 0.2172 | 0.3116 | 0.4857 | East Asian |
| Serum Mo | DKD | rs1504607 | C | T | 0.3301 | 0.0698 | 2E-06 | 22.39 | C | T | -0.2791 | 0.3216 | 0.3855 | East Asian |
| Serum Mo | DKD | rs39797 | C | T | -0.3809 | 0.0752 | 5E-07 | 25.66 | C | T | 0.3031 | 0.2505 | 0.2263 | East Asian |
| Serum Mo | DKD | rs75943454 | G | A | 0.4397 | 0.091 | 1E-06 | 23.37 | G | A | 0.074 | 0.2236 | 0.7406 | East Asian |
| Serum Cd | DKD | rs12228069 | A | G | -0.7078 | 0.1526 | 4E-06 | 21.52 | A | G | -0.0954 | 0.3292 | 0.7719 | East Asian |
| Serum Cd | DKD | rs166722 | G | A | -0.1707 | 0.0349 | 1E-06 | 24.00 | G | A | 0.0551 | 0.104 | 0.5966 | East Asian |
| Serum Cd | DKD | rs396511 | A | C | 0.2686 | 0.0586 | 5E-06 | 21.02 | A | C | 0.1226 | 0.1712 | 0.4741 | East Asian |
| Serum Cd | DKD | rs79052248 | G | A | 0.2285 | 0.0469 | 1E-06 | 23.76 | G | A | -0.0529 | 0.1316 | 0.6875 | East Asian |
| Serum Cd | DKD | rs880423 | C | T | 0.1588 | 0.0342 | 4E-06 | 21.58 | C | T | -0.0972 | 0.1008 | 0.3349 | East Asian |
| Serum V | DKD | rs2083833 | T | C | 0.1909 | 0.0415 | 4E-06 | 21.19 | T | C | -0.009 | 0.11 | 0.935 | East Asian |
| Serum V | DKD | rs249287 | G | T | 0.1651 | 0.0354 | 3E-06 | 21.77 | G | T | -0.0471 | 0.1047 | 0.6529 | East Asian |
| Serum V | DKD | rs534159 | T | C | -0.1866 | 0.0395 | 3E-06 | 22.29 | T | C | -0.0654 | 0.1052 | 0.5343 | East Asian |
| Serum V | DKD | rs6936473 | C | T | -0.1754 | 0.0375 | 3E-06 | 21.89 | C | T | -0.0843 | 0.1101 | 0.4439 | East Asian |
| Serum V | DKD | rs7282078 | C | T | -0.1887 | 0.039 | 1E-06 | 23.35 | C | T | -0.0162 | 0.1132 | 0.8862 | East Asian |
| Serum V | DKD | rs79070055 | T | C | -0.3515 | 0.0754 | 3E-06 | 21.71 | T | C | 0.5989 | 0.4387 | 0.1722 | East Asian |
| Serum V | DKD | rs972738 | G | A | -0.17 | 0.0334 | 4E-07 | 25.89 | G | A | -0.0794 | 0.0945 | 0.4006 | East Asian |
| Serum Cr | DKD | rs117244450 | A | C | 0.2204 | 0.0469 | 3E-06 | 22.12 | A | C | -0.0323 | 0.1427 | 0.8211 | East Asian |
| Serum Cr | DKD | rs12602392 | T | C | 0.1697 | 0.037 | 5E-06 | 21.04 | T | C | -0.0837 | 0.0993 | 0.3994 | East Asian |
| Serum Cr | DKD | rs12803936 | T | C | -0.1916 | 0.0404 | 2E-06 | 22.49 | T | C | -0.078 | 0.1259 | 0.5355 | East Asian |
| Serum Cr | DKD | rs61924870 | G | A | -0.3532 | 0.0727 | 1E-06 | 23.59 | G | A | -0.0702 | 0.2945 | 0.8115 | East Asian |
| Serum Cr | DKD | rs9487211 | C | T | -0.1768 | 0.0358 | 9E-07 | 24.40 | C | T | -0.1252 | 0.1112 | 0.2601 | East Asian |
| Serum Al | DKD | rs1948745 | A | G | 0.2478 | 0.0523 | 2E-06 | 22.45 | A | G | -0.0542 | 0.117 | 0.6434 | East Asian |
| Serum Al | DKD | rs4574233 | T | C | 0.2309 | 0.0475 | 1E-06 | 23.61 | T | C | 0.2483 | 0.1226 | 0.0429 | East Asian |
| Serum Al | DKD | rs4878080 | A | G | 0.1823 | 0.036 | 5E-07 | 25.58 | A | G | -0.0929 | 0.1147 | 0.4178 | East Asian |
| Serum Mn | DKD | rs149628902 | C | T | 0.3801 | 0.0815 | 3E-06 | 21.73 | C | T | 0.2662 | 0.4594 | 0.5623 | East Asian |
| Serum Mn | DKD | rs2155533 | A | G | 0.2018 | 0.0417 | 1E-06 | 23.42 | A | G | 0.084 | 0.1121 | 0.4538 | East Asian |
| Serum Ni | DKD | rs10772352 | G | A | 0.1864 | 0.0388 | 2E-06 | 23.09 | G | A | -0.067 | 0.1094 | 0.5406 | East Asian |
| Serum Ni | DKD | rs114018631 | T | C | 0.7646 | 0.159 | 2E-06 | 23.12 | T | C | -0.7651 | 0.4814 | 0.112 | East Asian |
| Serum Ni | DKD | rs12403087 | A | G | 0.1667 | 0.0359 | 4E-06 | 21.61 | A | G | 0.1812 | 0.0977 | 0.0637 | East Asian |
| Serum Ni | DKD | rs145980678 | A | G | -0.5668 | 0.1169 | 1E-06 | 23.52 | A | G | 0.5334 | 0.3211 | 0.0967 | East Asian |
| Serum Ni | DKD | rs2703457 | C | T | -0.2715 | 0.0583 | 4E-06 | 21.65 | C | T | 0.1944 | 0.1401 | 0.1654 | East Asian |
| Serum Ni | DKD | rs6035716 | T | C | 0.2839 | 0.0616 | 4E-06 | 21.27 | T | C | 0.2952 | 0.1692 | 0.081 | East Asian |
| Serum Ni | DKD | rs60926986 | C | T | 0.1785 | 0.0363 | 1E-06 | 24.15 | C | T | 0.0994 | 0.1038 | 0.3381 | East Asian |
| Serum Ni | DKD | rs7713992 | A | C | -0.1624 | 0.0347 | 3E-06 | 21.85 | A | C | 0.009 | 0.0964 | 0.9254 | East Asian |
| Serum Sn | DKD | rs2595886 | A | C | -0.2668 | 0.056 | 2E-06 | 22.72 | A | C | -0.3877 | 0.2314 | 0.0938 | East Asian |
| Serum Ti | DKD | rs17007550 | T | C | 0.1689 | 0.0358 | 3E-06 | 22.24 | T | C | 0.1412 | 0.0969 | 0.1451 | East Asian |
| Serum Ti | DKD | rs1843704 | T | C | 0.2319 | 0.0494 | 3E-06 | 22.07 | T | C | 0.0802 | 0.1398 | 0.5659 | East Asian |
| Serum Ti | DKD | rs6990315 | A | G | 0.4762 | 0.1014 | 3E-06 | 22.05 | A | G | -0.093 | 0.173 | 0.591 | East Asian |
| Serum Ti | DKD | rs72663511 | C | T | 0.3505 | 0.0738 | 2E-06 | 22.53 | C | T | -0.0633 | 0.1718 | 0.7125 | East Asian |
| Serum Ti | DKD | rs9932904 | C | T | -0.1629 | 0.0325 | 6E-07 | 25.18 | C | T | -0.013 | 0.0963 | 0.893 | East Asian |
| Serum Rb | DKD | rs10947698 | A | G | -0.321 | 0.0673 | 2E-06 | 22.74 | A | G | 0.0381 | 0.1326 | 0.7738 | East Asian |
| Serum Rb | DKD | rs1432288 | T | G | -0.1697 | 0.037 | 5E-06 | 21.02 | T | G | 0.0543 | 0.1101 | 0.6218 | East Asian |
| Serum Rb | DKD | rs2454029 | T | C | 0.2337 | 0.0499 | 3E-06 | 21.96 | T | C | -0.0967 | 0.1645 | 0.5564 | East Asian |
| Serum Rb | DKD | rs61273049 | A | G | 0.6914 | 0.1429 | 1E-06 | 23.40 | A | G | -0.0203 | 0.314 | 0.9484 | East Asian |
| Serum Sr | DKD | rs11897623 | C | T | -0.4271 | 0.079 | 7E-08 | 29.23 | C | T | 0.0156 | 0.2236 | 0.9445 | East Asian |
| Serum Sr | DKD | rs145046785 | A | G | 0.4165 | 0.0907 | 5E-06 | 21.08 | A | G | 0.0794 | 0.281 | 0.7775 | East Asian |
| Serum Sr | DKD | rs2289765 | G | A | 0.1855 | 0.0354 | 2E-07 | 27.46 | G | A | 0.0378 | 0.1179 | 0.7488 | East Asian |
| Serum Sr | DKD | rs2962476 | C | T | 0.2156 | 0.0459 | 3E-06 | 22.06 | C | T | -0.1584 | 0.1268 | 0.2116 | East Asian |
| Serum Sr | DKD | rs4707047 | T | C | -0.1589 | 0.0335 | 2E-06 | 22.53 | T | C | -0.1022 | 0.1089 | 0.3484 | East Asian |
| Serum Sr | DKD | rs6580219 | G | A | 0.1638 | 0.0354 | 4E-06 | 21.46 | G | A | -0.0006 | 0.1052 | 0.9958 | East Asian |
| Serum Sr | DKD | rs7090929 | C | A | 0.1783 | 0.0365 | 1E-06 | 23.87 | C | A | 0.2704 | 0.1346 | 0.0445 | East Asian |
| Serum Sr | DKD | rs74696940 | G | T | 0.3916 | 0.0827 | 2E-06 | 22.44 | G | T | -0.9329 | 0.4176 | 0.0255 | East Asian |
| Serum Cu | DKD | rs10424895 | A | G | 0.1978 | 0.036 | 4E-08 | 30.22 | A | G | -0.044 | 0.096 | 0.6471 | East Asian |
| Serum Cu | DKD | rs11708215 | G | A | 0.1734 | 0.035 | 8E-07 | 24.55 | G | A | 0.0505 | 0.1027 | 0.6231 | East Asian |
| Serum Cu | DKD | rs145001356 | T | C | -0.4297 | 0.0909 | 2E-06 | 22.36 | T | C | 0.18 | 0.2479 | 0.4678 | East Asian |
| Serum Cu | DKD | rs2857658 | A | G | 0.2531 | 0.0552 | 5E-06 | 21.01 | A | G | -0.0751 | 0.1911 | 0.6944 | East Asian |
| Serum Cu | DKD | rs60384721 | T | C | 0.1714 | 0.0371 | 4E-06 | 21.31 | T | C | 0.0708 | 0.1039 | 0.4957 | East Asian |
| Serum Cu | DKD | rs78069066 | A | G | -0.2399 | 0.0404 | 4E-09 | 35.19 | A | G | -0.2313 | 0.1122 | 0.0393 | East Asian |
| Serum Zn | DKD | rs17152037 | C | T | -0.1828 | 0.0381 | 2E-06 | 23.00 | C | T | 0.0083 | 0.1034 | 0.9361 | East Asian |
| Serum Zn | DKD | rs4272884 | C | A | -0.189 | 0.0402 | 3E-06 | 22.05 | C | A | -0.0413 | 0.1053 | 0.6945 | East Asian |
| Serum Zn | DKD | rs6766011 | G | A | -0.2063 | 0.0433 | 2E-06 | 22.74 | G | A | 0.1747 | 0.1165 | 0.1337 | East Asian |
| Serum Zn | DKD | rs6773812 | C | T | -0.1581 | 0.0321 | 9E-07 | 24.30 | C | T | 0.001 | 0.0952 | 0.9917 | East Asian |
| Serum Zn | DKD | rs79142685 | C | T | -0.2144 | 0.0431 | 7E-07 | 24.69 | C | T | -0.0634 | 0.164 | 0.699 | East Asian |
| Plasma Ba | DKD | rs11696219 | T | C | 0.3485 | 0.0705 | 1E-06 | 24.42 | T | C | -0.0559 | 0.1115 | 0.6165 | East Asian |
| Plasma Ba | DKD | rs183939842 | A | G | -1.0129 | 0.2188 | 4E-06 | 21.43 | A | G | -1.0588 | 1.1534 | 0.3586 | East Asian |
| Plasma Ba | DKD | rs2828460 | C | T | 0.3094 | 0.062 | 8E-07 | 24.94 | C | T | -0.0211 | 0.1245 | 0.8656 | East Asian |
| Plasma Ba | DKD | rs3017937 | G | A | 0.4574 | 0.0977 | 3E-06 | 21.91 | G | A | 0.1562 | 0.2138 | 0.465 | East Asian |
| Plasma Ba | DKD | rs35900478 | A | C | -0.3167 | 0.0674 | 3E-06 | 22.11 | A | C | -0.037 | 0.1017 | 0.7159 | East Asian |
| Plasma Ba | DKD | rs58174206 | C | T | -0.4302 | 0.0923 | 4E-06 | 21.71 | C | T | -0.0223 | 0.137 | 0.8705 | East Asian |
| Plasma Ba | DKD | rs80272920 | G | A | -0.7007 | 0.1522 | 5E-06 | 21.20 | G | A | -0.0533 | 0.4008 | 0.8941 | East Asian |
| Plasma Ba | DKD | rs9981507 | T | G | -0.6534 | 0.1406 | 4E-06 | 21.61 | T | G | -0.3082 | 0.3386 | 0.3627 | East Asian |
| Plasma Pb | DKD | rs2744386 | T | C | 0.5322 | 0.1124 | 3E-06 | 22.43 | T | C | 0.1862 | 0.1558 | 0.232 | East Asian |
| Plasma Pb | DKD | rs4237391 | T | G | 0.276 | 0.0561 | 1E-06 | 24.17 | T | G | -0.0351 | 0.0952 | 0.7129 | East Asian |
| Plasma Co | DKD | rs11772660 | G | A | -0.3835 | 0.0802 | 2E-06 | 22.88 | G | A | 0.0218 | 0.1373 | 0.8738 | East Asian |
| Plasma Co | DKD | rs2727867 | A | G | 0.2912 | 0.0597 | 1E-06 | 23.83 | A | G | 0.0516 | 0.0946 | 0.5856 | East Asian |
| Plasma Co | DKD | rs7103346 | A | G | 0.2477 | 0.0533 | 4E-06 | 21.56 | A | G | 0.0819 | 0.1071 | 0.4443 | East Asian |
| Plasma Mo | DKD | rs75597017 | G | A | -0.5709 | 0.124 | 5E-06 | 21.20 | G | A | -0.022 | 0.1835 | 0.9045 | East Asian |
| Plasma Mo | DKD | rs8021455 | C | T | -0.3963 | 0.0814 | 1E-06 | 23.70 | C | T | 0.16 | 0.1792 | 0.372 | East Asian |
| Plasma Cd | DKD | rs116449108 | T | C | 0.4357 | 0.0942 | 5E-06 | 21.40 | T | C | -0.1354 | 0.2169 | 0.5326 | East Asian |
| Plasma Cd | DKD | rs117609103 | G | A | 0.9274 | 0.1992 | 4E-06 | 21.67 | G | A | 0.2014 | 0.1808 | 0.2654 | East Asian |
| Plasma Cd | DKD | rs7124398 | G | T | -0.5844 | 0.1109 | 2E-07 | 27.77 | G | T | 0.1837 | 0.147 | 0.2115 | East Asian |
| Plasma Cd | DKD | rs79898619 | A | C | -0.4144 | 0.0819 | 5E-07 | 25.61 | A | C | -0.4503 | 0.1935 | 0.0199 | East Asian |
| Plasma V | DKD | rs4544187 | T | C | -0.3393 | 0.0627 | 9E-08 | 29.27 | T | C | 0.1484 | 0.0976 | 0.1282 | East Asian |
| Plasma V | DKD | rs6026593 | G | A | 0.3854 | 0.0798 | 2E-06 | 23.31 | G | A | 0.0101 | 0.1193 | 0.9324 | East Asian |
| Plasma Cr | DKD | rs11017755 | T | C | -0.3078 | 0.0634 | 1E-06 | 23.60 | T | C | 0.1495 | 0.1483 | 0.3135 | East Asian |
| Plasma Cr | DKD | rs12607014 | T | C | -0.3418 | 0.0637 | 1E-07 | 28.81 | T | C | 0.0079 | 0.1029 | 0.9392 | East Asian |
| Plasma Cr | DKD | rs62234189 | T | C | -0.9752 | 0.179 | 7E-08 | 29.69 | T | C | -0.3458 | 0.1782 | 0.0523 | East Asian |
| Plasma Al | DKD | rs1289661 | C | T | -0.3572 | 0.0767 | 4E-06 | 21.69 | C | T | -0.1742 | 0.1261 | 0.1672 | East Asian |
| Plasma Al | DKD | rs149814693 | A | G | 1.2045 | 0.2436 | 1E-06 | 24.45 | A | G | -0.3004 | 0.297 | 0.3117 | East Asian |
| Plasma Mn | DKD | rs61945916 | A | C | -0.5728 | 0.1186 | 2E-06 | 23.32 | A | C | -0.4747 | 0.2602 | 0.0681 | East Asian |
| Plasma Ni | DKD | rs12975867 | C | T | -0.4953 | 0.1057 | 3E-06 | 21.96 | C | T | 0.0164 | 0.1928 | 0.9321 | East Asian |
| Plasma Ni | DKD | rs144999307 | A | G | 0.5752 | 0.1177 | 1E-06 | 23.89 | A | G | 0.069 | 0.2195 | 0.7531 | East Asian |
| Plasma Ni | DKD | rs183518940 | A | G | 0.7353 | 0.1437 | 4E-07 | 26.19 | A | G | 1.7626 | 0.8063 | 0.0288 | East Asian |
| Plasma Ni | DKD | rs35786473 | T | C | -0.2867 | 0.061 | 3E-06 | 22.06 | T | C | 0.1259 | 0.1008 | 0.212 | East Asian |
| Plasma Ni | DKD | rs7201673 | T | C | 0.2993 | 0.0649 | 5E-06 | 21.27 | T | C | -0.0064 | 0.1033 | 0.9509 | East Asian |
| Plasma Sn | DKD | rs144086039 | G | A | 1.0128 | 0.2179 | 4E-06 | 21.61 | G | A | 0.7854 | 0.7772 | 0.3122 | East Asian |
| Plasma Ti | DKD | rs151209811 | T | C | -1.0181 | 0.2211 | 5E-06 | 21.20 | T | C | -0.2041 | 0.3575 | 0.568 | East Asian |
| Plasma Ti | DKD | rs355020 | A | G | -0.3376 | 0.0674 | 7E-07 | 25.08 | A | G | -0.2374 | 0.0949 | 0.0123 | East Asian |
| Plasma Ti | DKD | rs36110069 | G | T | 0.5999 | 0.118 | 5E-07 | 25.84 | G | T | -0.4177 | 0.171 | 0.0146 | East Asian |
| Plasma Rb | DKD | rs118182737 | A | G | 0.5894 | 0.118 | 8E-07 | 24.95 | A | G | 0.3309 | 0.2612 | 0.2052 | East Asian |
| Plasma Rb | DKD | rs146822775 | G | A | 0.4849 | 0.101 | 2E-06 | 23.04 | G | A | 0.0856 | 0.3277 | 0.794 | East Asian |
| Plasma Sr | DKD | rs10014591 | C | A | -0.3121 | 0.0674 | 4E-06 | 21.42 | C | A | 0.0848 | 0.119 | 0.4761 | East Asian |
| Plasma Sr | DKD | rs112043376 | A | C | 0.7791 | 0.1669 | 4E-06 | 21.78 | A | C | -0.2023 | 0.3326 | 0.543 | East Asian |
| Plasma Cu | DKD | rs17720055 | C | T | 0.4134 | 0.0848 | 1E-06 | 23.78 | C | T | -0.0842 | 0.1938 | 0.664 | East Asian |
| Plasma Cu | DKD | rs185903133 | A | G | -0.7239 | 0.1564 | 4E-06 | 21.43 | A | G | -0.2811 | 0.2886 | 0.33 | East Asian |
| Plasma Cu | DKD | rs7736204 | A | G | -0.2586 | 0.0559 | 5E-06 | 21.39 | A | G | 0.1081 | 0.0995 | 0.2772 | East Asian |
| Plasma Zn | DKD | rs10399884 | A | G | -0.54 | 0.1169 | 5E-06 | 21.34 | A | G | -0.1497 | 0.1367 | 0.2736 | East Asian |
| Plasma Zn | DKD | rs1289661 | C | T | -0.3579 | 0.0761 | 3E-06 | 22.10 | C | T | -0.1742 | 0.1261 | 0.1672 | East Asian |
| Plasma Zn | DKD | rs55908498 | A | G | -0.3856 | 0.0827 | 4E-06 | 21.71 | A | G | -0.0838 | 0.1744 | 0.6309 | East Asian |
| DKD | Serum Ba | rs12070058 | A | T | 1.1862 | 0.2499 | 2E-06 | 22.54 | A | T | 0.0522 | 0.074 | 0.4801 | East Asian |
| DKD | Serum Ba | rs12214931 | G | T | 0.586 | 0.1214 | 1E-06 | 23.30 | G | T | -0.0099 | 0.0419 | 0.8137 | East Asian |
| DKD | Serum Ba | rs375463535 | G | A | 31.426 | 6.4782 | 1E-06 | 23.53 | G | A | -0.0431 | 0.1301 | 0.7405 | East Asian |
| DKD | Serum Ba | rs6552912 | G | A | 0.5687 | 0.1196 | 2E-06 | 22.60 | G | A | -0.0009 | 0.044 | 0.9833 | East Asian |
| DKD | Serum Pb | rs12070058 | A | T | 1.1862 | 0.2499 | 2E-06 | 22.54 | A | T | 0.0226 | 0.0743 | 0.7606 | East Asian |
| DKD | Serum Pb | rs12214931 | G | T | 0.586 | 0.1214 | 1E-06 | 23.30 | G | T | -0.0441 | 0.0418 | 0.2912 | East Asian |
| DKD | Serum Pb | rs375463535 | G | A | 31.426 | 6.4782 | 1E-06 | 23.53 | G | A | 0.0169 | 0.1287 | 0.8953 | East Asian |
| DKD | Serum Pb | rs6552912 | G | A | 0.5687 | 0.1196 | 2E-06 | 22.60 | G | A | 0.0552 | 0.0437 | 0.2066 | East Asian |
| DKD | Serum Co | rs12070058 | A | T | 1.1862 | 0.2499 | 2E-06 | 22.54 | A | T | -0.1005 | 0.0755 | 0.1833 | East Asian |
| DKD | Serum Co | rs12214931 | G | T | 0.586 | 0.1214 | 1E-06 | 23.30 | G | T | 0.0367 | 0.0424 | 0.3861 | East Asian |
| DKD | Serum Co | rs375463535 | G | A | 31.426 | 6.4782 | 1E-06 | 23.53 | G | A | 0.0509 | 0.1318 | 0.6992 | East Asian |
| DKD | Serum Co | rs6552912 | G | A | 0.5687 | 0.1196 | 2E-06 | 22.60 | G | A | 0.0332 | 0.0448 | 0.4579 | East Asian |
| DKD | Serum Mo | rs12070058 | A | T | 1.1862 | 0.2499 | 2E-06 | 22.54 | A | T | 0.0116 | 0.075 | 0.8767 | East Asian |
| DKD | Serum Mo | rs12214931 | G | T | 0.586 | 0.1214 | 1E-06 | 23.30 | G | T | 0.046 | 0.042 | 0.2737 | East Asian |
| DKD | Serum Mo | rs375463535 | G | A | 31.426 | 6.4782 | 1E-06 | 23.53 | G | A | -0.1126 | 0.1305 | 0.3883 | East Asian |
| DKD | Serum Mo | rs6552912 | G | A | 0.5687 | 0.1196 | 2E-06 | 22.60 | G | A | -0.0094 | 0.0442 | 0.8307 | East Asian |
| DKD | Serum Cd | rs12070058 | A | T | 1.1862 | 0.2499 | 2E-06 | 22.54 | A | T | 0.0731 | 0.0683 | 0.2848 | East Asian |
| DKD | Serum Cd | rs12214931 | G | T | 0.586 | 0.1214 | 1E-06 | 23.30 | G | T | 0.042 | 0.0388 | 0.2794 | East Asian |
| DKD | Serum Cd | rs375463535 | G | A | 31.426 | 6.4782 | 1E-06 | 23.53 | G | A | -0.2079 | 0.1235 | 0.0923 | East Asian |
| DKD | Serum Cd | rs6552912 | G | A | 0.5687 | 0.1196 | 2E-06 | 22.60 | G | A | 0.0076 | 0.0406 | 0.8522 | East Asian |
| DKD | Serum V | rs12070058 | A | T | 1.1862 | 0.2499 | 2E-06 | 22.54 | A | T | 0.0717 | 0.0741 | 0.3329 | East Asian |
| DKD | Serum V | rs12214931 | G | T | 0.586 | 0.1214 | 1E-06 | 23.30 | G | T | -0.0581 | 0.0418 | 0.1651 | East Asian |
| DKD | Serum V | rs375463535 | G | A | 31.426 | 6.4782 | 1E-06 | 23.53 | G | A | -0.1737 | 0.1303 | 0.1827 | East Asian |
| DKD | Serum V | rs6552912 | G | A | 0.5687 | 0.1196 | 2E-06 | 22.60 | G | A | -0.0007 | 0.0439 | 0.987 | East Asian |
| DKD | Serum Cr | rs12070058 | A | T | 1.1862 | 0.2499 | 2E-06 | 22.54 | A | T | -0.0428 | 0.0764 | 0.5752 | East Asian |
| DKD | Serum Cr | rs12214931 | G | T | 0.586 | 0.1214 | 1E-06 | 23.30 | G | T | 0.0162 | 0.043 | 0.7056 | East Asian |
| DKD | Serum Cr | rs375463535 | G | A | 31.426 | 6.4782 | 1E-06 | 23.53 | G | A | -0.0153 | 0.1344 | 0.9094 | East Asian |
| DKD | Serum Cr | rs6552912 | G | A | 0.5687 | 0.1196 | 2E-06 | 22.60 | G | A | -0.0105 | 0.0452 | 0.8169 | East Asian |
| DKD | Serum Al | rs12070058 | A | T | 1.1862 | 0.2499 | 2E-06 | 22.54 | A | T | 0.0687 | 0.0748 | 0.3586 | East Asian |
| DKD | Serum Al | rs12214931 | G | T | 0.586 | 0.1214 | 1E-06 | 23.30 | G | T | 0.0687 | 0.0424 | 0.1054 | East Asian |
| DKD | Serum Al | rs375463535 | G | A | 31.426 | 6.4782 | 1E-06 | 23.53 | G | A | -0.0246 | 0.1306 | 0.8505 | East Asian |
| DKD | Serum Al | rs6552912 | G | A | 0.5687 | 0.1196 | 2E-06 | 22.60 | G | A | 0.041 | 0.0442 | 0.3536 | East Asian |
| DKD | Serum Mn | rs12070058 | A | T | 1.1862 | 0.2499 | 2E-06 | 22.54 | A | T | 0.0659 | 0.0776 | 0.3957 | East Asian |
| DKD | Serum Mn | rs12214931 | G | T | 0.586 | 0.1214 | 1E-06 | 23.30 | G | T | -0.0396 | 0.0435 | 0.3626 | East Asian |
| DKD | Serum Mn | rs375463535 | G | A | 31.426 | 6.4782 | 1E-06 | 23.53 | G | A | 0.0488 | 0.1353 | 0.7184 | East Asian |
| DKD | Serum Mn | rs6552912 | G | A | 0.5687 | 0.1196 | 2E-06 | 22.60 | G | A | -0.0361 | 0.0459 | 0.4318 | East Asian |
| DKD | Serum Ni | rs12070058 | A | T | 1.1862 | 0.2499 | 2E-06 | 22.54 | A | T | 0.162 | 0.0762 | 0.0335 | East Asian |
| DKD | Serum Ni | rs12214931 | G | T | 0.586 | 0.1214 | 1E-06 | 23.30 | G | T | 0.0261 | 0.0431 | 0.5453 | East Asian |
| DKD | Serum Ni | rs375463535 | G | A | 31.426 | 6.4782 | 1E-06 | 23.53 | G | A | -0.0318 | 0.1355 | 0.8143 | East Asian |
| DKD | Serum Ni | rs6552912 | G | A | 0.5687 | 0.1196 | 2E-06 | 22.60 | G | A | -0.0036 | 0.0451 | 0.9355 | East Asian |
| DKD | Serum Sn | rs12070058 | A | T | 1.1862 | 0.2499 | 2E-06 | 22.54 | A | T | 0.0331 | 0.0635 | 0.6022 | East Asian |
| DKD | Serum Sn | rs12214931 | G | T | 0.586 | 0.1214 | 1E-06 | 23.30 | G | T | -0.0443 | 0.0359 | 0.217 | East Asian |
| DKD | Serum Sn | rs375463535 | G | A | 31.426 | 6.4782 | 1E-06 | 23.53 | G | A | 0.0211 | 0.1117 | 0.8503 | East Asian |
| DKD | Serum Sn | rs6552912 | G | A | 0.5687 | 0.1196 | 2E-06 | 22.60 | G | A | -0.0585 | 0.0377 | 0.1208 | East Asian |
| DKD | Serum Ti | rs12070058 | A | T | 1.1862 | 0.2499 | 2E-06 | 22.54 | A | T | -0.0241 | 0.0732 | 0.7418 | East Asian |
| DKD | Serum Ti | rs12214931 | G | T | 0.586 | 0.1214 | 1E-06 | 23.30 | G | T | 0.027 | 0.0414 | 0.5136 | East Asian |
| DKD | Serum Ti | rs375463535 | G | A | 31.426 | 6.4782 | 1E-06 | 23.53 | G | A | 0.0866 | 0.1287 | 0.5009 | East Asian |
| DKD | Serum Ti | rs6552912 | G | A | 0.5687 | 0.1196 | 2E-06 | 22.60 | G | A | -0.0031 | 0.0434 | 0.9424 | East Asian |
| DKD | Serum Rb | rs12070058 | A | T | 1.1862 | 0.2499 | 2E-06 | 22.54 | A | T | 0.0135 | 0.0742 | 0.8558 | East Asian |
| DKD | Serum Rb | rs12214931 | G | T | 0.586 | 0.1214 | 1E-06 | 23.30 | G | T | 0.0519 | 0.0419 | 0.2152 | East Asian |
| DKD | Serum Rb | rs375463535 | G | A | 31.426 | 6.4782 | 1E-06 | 23.53 | G | A | 0.0861 | 0.1303 | 0.5088 | East Asian |
| DKD | Serum Rb | rs6552912 | G | A | 0.5687 | 0.1196 | 2E-06 | 22.60 | G | A | -0.0687 | 0.044 | 0.1183 | East Asian |
| DKD | Serum Sr | rs12070058 | A | T | 1.1862 | 0.2499 | 2E-06 | 22.54 | A | T | 0.0339 | 0.0695 | 0.6255 | East Asian |
| DKD | Serum Sr | rs12214931 | G | T | 0.586 | 0.1214 | 1E-06 | 23.30 | G | T | -0.0003 | 0.0394 | 0.9949 | East Asian |
| DKD | Serum Sr | rs375463535 | G | A | 31.426 | 6.4782 | 1E-06 | 23.53 | G | A | -0.017 | 0.1222 | 0.8892 | East Asian |
| DKD | Serum Sr | rs6552912 | G | A | 0.5687 | 0.1196 | 2E-06 | 22.60 | G | A | 0.0059 | 0.0412 | 0.8865 | East Asian |
| DKD | Serum Cu | rs12070058 | A | T | 1.1862 | 0.2499 | 2E-06 | 22.54 | A | T | -0.037 | 0.0738 | 0.6165 | East Asian |
| DKD | Serum Cu | rs12214931 | G | T | 0.586 | 0.1214 | 1E-06 | 23.30 | G | T | 0.0258 | 0.0417 | 0.5357 | East Asian |
| DKD | Serum Cu | rs375463535 | G | A | 31.426 | 6.4782 | 1E-06 | 23.53 | G | A | 0.0633 | 0.1298 | 0.6258 | East Asian |
| DKD | Serum Cu | rs6552912 | G | A | 0.5687 | 0.1196 | 2E-06 | 22.60 | G | A | -0.0012 | 0.0437 | 0.9786 | East Asian |
| DKD | Serum Zn | rs12070058 | A | T | 1.1862 | 0.2499 | 2E-06 | 22.54 | A | T | 0.0953 | 0.0737 | 0.1966 | East Asian |
| DKD | Serum Zn | rs12214931 | G | T | 0.586 | 0.1214 | 1E-06 | 23.30 | G | T | -0.0101 | 0.0416 | 0.8077 | East Asian |
| DKD | Serum Zn | rs375463535 | G | A | 31.426 | 6.4782 | 1E-06 | 23.53 | G | A | -0.015 | 0.1295 | 0.9076 | East Asian |
| DKD | Serum Zn | rs6552912 | G | A | 0.5687 | 0.1196 | 2E-06 | 22.60 | G | A | -0.045 | 0.0436 | 0.3024 | East Asian |
| DKD | Plasma Ba | rs12070058 | A | T | 1.1862 | 0.2499 | 2E-06 | 22.54 | A | T | -0.2267 | 0.1126 | 0.0445 | East Asian |
| DKD | Plasma Ba | rs12214931 | G | T | 0.586 | 0.1214 | 1E-06 | 23.30 | G | T | 0.043 | 0.0839 | 0.6084 | East Asian |
| DKD | Plasma Ba | rs6552912 | G | A | 0.5687 | 0.1196 | 2E-06 | 22.60 | G | A | 0.089 | 0.0705 | 0.2068 | East Asian |
| DKD | Plasma Pb | rs12070058 | A | T | 1.1862 | 0.2499 | 2E-06 | 22.54 | A | T | -0.2056 | 0.1058 | 0.0524 | East Asian |
| DKD | Plasma Pb | rs12214931 | G | T | 0.586 | 0.1214 | 1E-06 | 23.30 | G | T | 0.1036 | 0.0789 | 0.1895 | East Asian |
| DKD | Plasma Pb | rs6552912 | G | A | 0.5687 | 0.1196 | 2E-06 | 22.60 | G | A | 0.0114 | 0.0666 | 0.8644 | East Asian |
| DKD | Plasma Co | rs12070058 | A | T | 1.1862 | 0.2499 | 2E-06 | 22.54 | A | T | -0.1641 | 0.108 | 0.1291 | East Asian |
| DKD | Plasma Co | rs12214931 | G | T | 0.586 | 0.1214 | 1E-06 | 23.30 | G | T | 0.0619 | 0.082 | 0.45 | East Asian |
| DKD | Plasma Co | rs6552912 | G | A | 0.5687 | 0.1196 | 2E-06 | 22.60 | G | A | -0.0184 | 0.0674 | 0.7851 | East Asian |
| DKD | Plasma Mo | rs12070058 | A | T | 1.1862 | 0.2499 | 2E-06 | 22.54 | A | T | -0.0123 | 0.1118 | 0.9121 | East Asian |
| DKD | Plasma Mo | rs12214931 | G | T | 0.586 | 0.1214 | 1E-06 | 23.30 | G | T | -0.1876 | 0.083 | 0.0242 | East Asian |
| DKD | Plasma Mo | rs6552912 | G | A | 0.5687 | 0.1196 | 2E-06 | 22.60 | G | A | 0.0883 | 0.0699 | 0.207 | East Asian |
| DKD | Plasma Cd | rs12070058 | A | T | 1.1862 | 0.2499 | 2E-06 | 22.54 | A | T | -0.0333 | 0.1158 | 0.7739 | East Asian |
| DKD | Plasma Cd | rs12214931 | G | T | 0.586 | 0.1214 | 1E-06 | 23.30 | G | T | 0.1076 | 0.0864 | 0.2137 | East Asian |
| DKD | Plasma Cd | rs6552912 | G | A | 0.5687 | 0.1196 | 2E-06 | 22.60 | G | A | -0.0581 | 0.0732 | 0.4275 | East Asian |
| DKD | Plasma V | rs12070058 | A | T | 1.1862 | 0.2499 | 2E-06 | 22.54 | A | T | 0.1384 | 0.1142 | 0.226 | East Asian |
| DKD | Plasma V | rs12214931 | G | T | 0.586 | 0.1214 | 1E-06 | 23.30 | G | T | -0.0219 | 0.0849 | 0.7962 | East Asian |
| DKD | Plasma V | rs6552912 | G | A | 0.5687 | 0.1196 | 2E-06 | 22.60 | G | A | 0.0276 | 0.0714 | 0.6993 | East Asian |
| DKD | Plasma Cr | rs12070058 | A | T | 1.1862 | 0.2499 | 2E-06 | 22.54 | A | T | -0.0302 | 0.1136 | 0.7905 | East Asian |
| DKD | Plasma Cr | rs12214931 | G | T | 0.586 | 0.1214 | 1E-06 | 23.30 | G | T | 0.015 | 0.0841 | 0.8583 | East Asian |
| DKD | Plasma Cr | rs6552912 | G | A | 0.5687 | 0.1196 | 2E-06 | 22.60 | G | A | -0.0358 | 0.0708 | 0.6136 | East Asian |
| DKD | Plasma Al | rs12070058 | A | T | 1.1862 | 0.2499 | 2E-06 | 22.54 | A | T | -0.0682 | 0.1149 | 0.553 | East Asian |
| DKD | Plasma Al | rs12214931 | G | T | 0.586 | 0.1214 | 1E-06 | 23.30 | G | T | -0.0321 | 0.0851 | 0.7062 | East Asian |
| DKD | Plasma Al | rs6552912 | G | A | 0.5687 | 0.1196 | 2E-06 | 22.60 | G | A | 0.1237 | 0.0711 | 0.0826 | East Asian |
| DKD | Plasma Mn | rs12070058 | A | T | 1.1862 | 0.2499 | 2E-06 | 22.54 | A | T | -0.1374 | 0.1131 | 0.225 | East Asian |
| DKD | Plasma Mn | rs12214931 | G | T | 0.586 | 0.1214 | 1E-06 | 23.30 | G | T | -0.0163 | 0.0844 | 0.8468 | East Asian |
| DKD | Plasma Mn | rs6552912 | G | A | 0.5687 | 0.1196 | 2E-06 | 22.60 | G | A | 0.0412 | 0.0711 | 0.5626 | East Asian |
| DKD | Plasma Ni | rs12070058 | A | T | 1.1862 | 0.2499 | 2E-06 | 22.54 | A | T | -0.2242 | 0.1115 | 0.0448 | East Asian |
| DKD | Plasma Ni | rs12214931 | G | T | 0.586 | 0.1214 | 1E-06 | 23.30 | G | T | 0.049 | 0.0831 | 0.556 | East Asian |
| DKD | Plasma Ni | rs6552912 | G | A | 0.5687 | 0.1196 | 2E-06 | 22.60 | G | A | 0.0697 | 0.0699 | 0.3191 | East Asian |
| DKD | Plasma Sn | rs12070058 | A | T | 1.1862 | 0.2499 | 2E-06 | 22.54 | A | T | 0.1573 | 0.1139 | 0.1678 | East Asian |
| DKD | Plasma Sn | rs12214931 | G | T | 0.586 | 0.1214 | 1E-06 | 23.30 | G | T | -0.137 | 0.0864 | 0.1134 | East Asian |
| DKD | Plasma Sn | rs6552912 | G | A | 0.5687 | 0.1196 | 2E-06 | 22.60 | G | A | -0.0733 | 0.0725 | 0.3126 | East Asian |
| DKD | Plasma Ti | rs12070058 | A | T | 1.1862 | 0.2499 | 2E-06 | 22.54 | A | T | 0.2798 | 0.1134 | 0.0139 | East Asian |
| DKD | Plasma Ti | rs12214931 | G | T | 0.586 | 0.1214 | 1E-06 | 23.30 | G | T | -0.081 | 0.0845 | 0.3383 | East Asian |
| DKD | Plasma Ti | rs6552912 | G | A | 0.5687 | 0.1196 | 2E-06 | 22.60 | G | A | -0.0019 | 0.0711 | 0.979 | East Asian |
| DKD | Plasma Rb | rs12070058 | A | T | 1.1862 | 0.2499 | 2E-06 | 22.54 | A | T | -0.256 | 0.1023 | 0.0125 | East Asian |
| DKD | Plasma Rb | rs12214931 | G | T | 0.586 | 0.1214 | 1E-06 | 23.30 | G | T | -0.0056 | 0.0763 | 0.9416 | East Asian |
| DKD | Plasma Rb | rs6552912 | G | A | 0.5687 | 0.1196 | 2E-06 | 22.60 | G | A | 0.0815 | 0.0641 | 0.2037 | East Asian |
| DKD | Plasma Sr | rs12070058 | A | T | 1.1862 | 0.2499 | 2E-06 | 22.54 | A | T | -0.0153 | 0.1134 | 0.8928 | East Asian |
| DKD | Plasma Sr | rs12214931 | G | T | 0.586 | 0.1214 | 1E-06 | 23.30 | G | T | -0.116 | 0.084 | 0.1678 | East Asian |
| DKD | Plasma Sr | rs6552912 | G | A | 0.5687 | 0.1196 | 2E-06 | 22.60 | G | A | -0.0555 | 0.0707 | 0.4331 | East Asian |
| DKD | Plasma Cu | rs12070058 | A | T | 1.1862 | 0.2499 | 2E-06 | 22.54 | A | T | -0.0576 | 0.1043 | 0.5808 | East Asian |
| DKD | Plasma Cu | rs12214931 | G | T | 0.586 | 0.1214 | 1E-06 | 23.30 | G | T | -0.0955 | 0.0775 | 0.2181 | East Asian |
| DKD | Plasma Cu | rs6552912 | G | A | 0.5687 | 0.1196 | 2E-06 | 22.60 | G | A | 0.0866 | 0.0652 | 0.1843 | East Asian |
| DKD | Plasma Zn | rs12070058 | A | T | 1.1862 | 0.2499 | 2E-06 | 22.54 | A | T | -0.1146 | 0.1136 | 0.3134 | East Asian |
| DKD | Plasma Zn | rs12214931 | G | T | 0.586 | 0.1214 | 1E-06 | 23.30 | G | T | 0.0551 | 0.0841 | 0.5128 | East Asian |
| DKD | Plasma Zn | rs6552912 | G | A | 0.5687 | 0.1196 | 2E-06 | 22.60 | G | A | 0.0053 | 0.0708 | 0.9407 | East Asian |
| Blood Al | DKD | rs138194016 | C | T | 1.5 | 0.291 | 3E-07 | 26.57 | C | T | -0.0953 | 0.239 | 0.69 | European |
| Blood Al | DKD | rs7521798 | C | T | 0.223 | 0.0469 | 2E-06 | 22.61 | C | T | 0.0202 | 0.0356 | 0.57 | European |
| Blood Al | DKD | rs75629201 | C | A | 0.755 | 0.148 | 3E-07 | 26.02 | C | A | 0.1054 | 0.075 | 0.16 | European |
| Blood Al | DKD | rs77795977 | C | T | -0.686 | 0.143 | 2E-06 | 23.01 | C | T | 0.0726 | 0.092 | 0.43 | European |
| Blood Cd | DKD | rs141495768 | A | G | 1.73 | 0.354 | 1E-06 | 23.88 | A | G | -0.2624 | 0.1867 | 0.16 | European |
| Blood Cd | DKD | rs143141392 | C | A | -1.22 | 0.25 | 9E-07 | 23.81 | C | A | -0.2624 | 0.1099 | 0.017 | European |
| Blood Cd | DKD | rs61839209 | T | C | 0.307 | 0.0622 | 8E-07 | 24.36 | T | C | 0.0202 | 0.0273 | 0.46 | European |
| Blood Cd | DKD | rs6948871 | C | T | 0.638 | 0.129 | 8E-07 | 24.46 | C | T | 0.1278 | 0.0844 | 0.13 | European |
| Blood Cd | DKD | rs79883490 | G | A | 0.403 | 0.0867 | 3E-06 | 21.61 | G | A | -0.0953 | 0.0939 | 0.31 | European |
| Blood Cd | DKD | rs9350504 | T | C | -1.16 | 0.18 | 1E-10 | 41.53 | T | C | -0.2624 | 0.2002 | 0.19 | European |
| Blood Cd | DKD | rs9875531 | A | G | 0.29 | 0.0625 | 4E-06 | 21.53 | A | G | 0.0513 | 0.0446 | 0.25 | European |
| Blood Co | DKD | rs12369605 | C | T | -1.29 | 0.264 | 9E-07 | 23.88 | C | T | -0.0953 | 0.2474 | 0.7 | European |
| Blood Co | DKD | rs55643745 | C | T | -1.02 | 0.221 | 4E-06 | 21.30 | C | T | 0.2231 | 0.1741 | 0.2 | European |
| Blood Co | DKD | rs6496588 | A | C | -0.474 | 0.102 | 3E-06 | 21.60 | A | C | 0.0202 | 0.0723 | 0.78 | European |
| Blood Cr | DKD | rs10784615 | A | G | -0.416 | 0.0884 | 3E-06 | 22.15 | A | G | 0.0408 | 0.0319 | 0.2 | European |
| Blood Cr | DKD | rs1079450 | T | C | -0.304 | 0.0645 | 2E-06 | 22.21 | T | C | 0.0101 | 0.0303 | 0.74 | European |
| Blood Cr | DKD | rs76797272 | C | T | -1.08 | 0.225 | 2E-06 | 23.04 | C | T | -0.0953 | 0.2659 | 0.72 | European |
| Blood Cu | DKD | rs11132992 | G | A | -0.304 | 0.0643 | 2E-06 | 22.35 | G | A | 0.0202 | 0.1461 | 0.89 | European |
| Blood Cu | DKD | rs1262685 | C | T | 1.11 | 0.238 | 3E-06 | 21.75 | C | T | -0.1823 | 0.1552 | 0.24 | European |
| Blood Cu | DKD | rs139127800 | A | G | -0.676 | 0.146 | 3E-06 | 21.44 | A | G | 0.0943 | 0.0786 | 0.23 | European |
| Blood Cu | DKD | rs141434375 | A | G | -1.03 | 0.208 | 9E-07 | 24.52 | A | G | 0.1165 | 0.0992 | 0.24 | European |
| Blood Cu | DKD | rs35746828 | A | G | -0.518 | 0.105 | 9E-07 | 24.34 | A | G | 0.0834 | 0.093 | 0.37 | European |
| Blood Cu | DKD | rs6127014 | G | A | -1.42 | 0.299 | 2E-06 | 22.55 | G | A | 0.0101 | 0.0498 | 0.84 | European |
| Blood Cu | DKD | rs76680160 | G | A | 0.675 | 0.129 | 2E-07 | 27.38 | G | A | 0.1508 | 0.0917 | 0.1 | European |
| Blood Cu | DKD | rs79497581 | G | A | -1.25 | 0.261 | 1E-06 | 22.94 | G | A | -0.0953 | 0.5404 | 0.86 | European |
| Blood Cu | DKD | rs9660771 | C | T | 0.217 | 0.0429 | 4E-07 | 25.59 | C | T | 0.0408 | 0.0236 | 0.084 | European |
| Blood Hg | DKD | rs114640911 | A | C | 0.987 | 0.216 | 5E-06 | 20.88 | A | C | 0.1744 | 0.13 | 0.18 | European |
| Blood Hg | DKD | rs72763586 | T | C | -0.403 | 0.0879 | 5E-06 | 21.02 | T | C | 0.0726 | 0.0658 | 0.27 | European |
| Blood Hg | DKD | rs78231031 | C | A | 1.33 | 0.283 | 3E-06 | 22.09 | C | A | 0.0305 | 0.0614 | 0.62 | European |
| Blood Mn | DKD | rs11006464 | T | C | 1.06 | 0.225 | 2E-06 | 22.19 | T | C | 0.1625 | 0.1634 | 0.32 | European |
| Blood Mn | DKD | rs147237795 | G | A | -1.17 | 0.243 | 2E-06 | 23.18 | G | A | -0.1823 | 0.2121 | 0.39 | European |
| Blood Mn | DKD | rs1776029 | A | G | -0.456 | 0.0596 | 2E-14 | 58.54 | A | G | 0.0202 | 0.0585 | 0.73 | European |
| Blood Mn | DKD | rs190511748 | G | A | -1.59 | 0.33 | 1E-06 | 23.21 | G | A | -0.2624 | 0.2047 | 0.2 | European |
| Blood Mn | DKD | rs2051166 | C | A | -0.419 | 0.0906 | 4E-06 | 21.39 | C | A | -0.0953 | 0.0571 | 0.095 | European |
| Blood Mn | DKD | rs6833623 | A | G | -0.428 | 0.0909 | 3E-06 | 22.17 | A | G | 0.0202 | 0.1608 | 0.9 | European |
| Blood Mo | DKD | rs11097881 | G | A | -0.344 | 0.0748 | 4E-06 | 21.15 | G | A | 0.0619 | 0.0676 | 0.36 | European |
| Blood Mo | DKD | rs72773218 | G | A | -1.67 | 0.328 | 3E-07 | 25.92 | G | A | 0.1278 | 0.1426 | 0.37 | European |
| Blood Mo | DKD | rs75888800 | G | A | 1.56 | 0.329 | 2E-06 | 22.48 | G | A | 0.0513 | 0.6815 | 0.94 | European |
| Blood Mo | DKD | rs77107990 | G | A | -0.413 | 0.0874 | 2E-06 | 22.33 | G | A | 0.0101 | 0.0418 | 0.81 | European |
| Blood Ni | DKD | rs111882271 | G | A | -0.814 | 0.154 | 1E-07 | 27.94 | G | A | -0.1823 | 0.1108 | 0.1 | European |
| Blood Ni | DKD | rs117702654 | A | G | -1.23 | 0.258 | 2E-06 | 22.73 | A | G | 0.1863 | 0.1486 | 0.21 | European |
| Blood Ni | DKD | rs137918052 | C | T | -1.29 | 0.256 | 5E-07 | 25.39 | C | T | 0.0305 | 0.0347 | 0.38 | European |
| Blood Ni | DKD | rs28491471 | G | T | -0.267 | 0.0574 | 3E-06 | 21.64 | G | T | -0.0953 | 0.0403 | 0.018 | European |
| Blood Ni | DKD | rs35216390 | T | C | -0.37 | 0.0755 | 1E-06 | 24.02 | T | C | 0.0408 | 0.0591 | 0.49 | European |
| Blood Ni | DKD | rs55759849 | A | G | -0.529 | 0.115 | 5E-06 | 21.16 | A | G | 0.0305 | 0.0565 | 0.59 | European |
| Blood Ni | DKD | rs7185243 | G | A | 1.06 | 0.227 | 3E-06 | 21.81 | G | A | 0.0305 | 0.0671 | 0.65 | European |
| Blood Ni | DKD | rs8040516 | T | C | 0.219 | 0.0468 | 3E-06 | 21.90 | T | C | 0.0101 | 0.1001 | 0.92 | European |
| Blood Pb | DKD | rs117121891 | C | A | 1.29 | 0.271 | 2E-06 | 22.66 | C | A | 0.0943 | 0.2733 | 0.73 | European |
| Blood Pb | DKD | rs1395910 | C | T | 0.218 | 0.0453 | 1E-06 | 23.16 | C | T | 0.0202 | 0.0407 | 0.62 | European |
| Blood Pb | DKD | rs2272278 | G | T | 0.241 | 0.0467 | 2E-07 | 26.63 | G | T | -0.0953 | 0.0466 | 0.041 | European |
| Blood Zn | DKD | rs10053875 | G | A | 0.74 | 0.16 | 4E-06 | 21.39 | G | A | -0.0953 | 0.1086 | 0.38 | European |
| Blood Zn | DKD | rs3104890 | T | C | 0.262 | 0.0562 | 3E-06 | 21.73 | T | C | 0.0726 | 0.0415 | 0.08 | European |
| Blood Zn | DKD | rs35390123 | T | G | 0.391 | 0.0783 | 6E-07 | 24.94 | T | G | 0.0513 | 0.0584 | 0.38 | European |
| Blood Zn | DKD | rs79479745 | A | G | -0.671 | 0.146 | 4E-06 | 21.12 | A | G | 0.1278 | 0.0822 | 0.12 | European |
| SNPs, single nucleotide polymorphisms; EA-E, effect allele of exposure; NEA-E, non-effect allele of exposure; Beta-E, beta of exposure; SE-E, standard error of exposure; *P*-E, the significance level of SNPs in exposure; EA-O, effect allele of outcome; NEA-O, non-effect allele of outcome; Beta-O, beta of outcome; SE-O, standard error of outcome; *P*-O, the significance level of SNPs in outcome. The *F* statistic for each SNP was calculated as follows: *F* = Beta^2^exposure/SE^2^exposure. | | | | | | | | | | | | | | |

**Table S3.** Mendelian randomization analysis results.

| **Exposure** | **Outcome** | **nSNPs** | **IVW^†^ or Wald ratio^‡^** | | |  | **Weighted median** | | |  | **MR Egger** | | |
| --- | --- | --- | --- | --- | --- | --- | --- | --- | --- | --- | --- | --- | --- |
|  |  |  | **OR^1^ / Beta^2^** | **95%CI^1^ / SE^2^** | ***P*-value** |  | **OR^1^ / Beta^2^** | **95%CI^1^ / SE^2^** | ***P*-value** |  | **OR^1^ / Beta^2^** | **95%CI^1^ / SE^2^** | ***P*-value** |
| Serum Ba | DKD | 5 | 0.910 | (0.49,1.69) | 0.754 |  | 0.970 | (0.52,1.82) | 0.933 |  | 0.880 | (0.10,7.58) | 0.914 |
| Serum Pb | DKD | 7 | 1.150 | (0.75,1.75) | 0.514 |  | 1.220 | (0.70,2.14) | 0.489 |  | 1.490 | (0.45,4.95) | 0.543 |
| Serum Co^†^ | DKD | 2 | 0.660 | (0.18,2.43) | 0.530 |  |  |  |  |  |  |  |  |
| Serum Mo | DKD | 5 | 1.110 | (0.62,1.99) | 0.731 |  | 1.320 | (0.61,2.88) | 0.479 |  | 0.490 | (0.11,2.26) | 0.429 |
| Serum Cd | DKD | 5 | 0.920 | (0.56,1.51) | 0.731 |  | 0.870 | (0.46,1.64) | 0.672 |  | 1.570 | (0.49,5.04) | 0.503 |
| Serum V | DKD | 7 | 1.130 | (0.71,1.79) | 0.614 |  | 1.230 | (0.71,2.13) | 0.466 |  | 0.070 | (0.00,5.07) | 0.275 |
| Serum Cr | DKD | 5 | 1.110 | (0.63,1.97) | 0.717 |  | 1.160 | (0.58,2.35) | 0.671 |  | 1.350 | (0.06,29.1) | 0.858 |
| Serum Al | DKD | 3 | 1.160 | (0.46,2.93) | 0.757 |  | 0.890 | (0.39,2.02) | 0.772 |  | 7.130 | (0.00,1e+5) | 0.757 |
| Serum Mn^†^ | DKD | 2 | 1.590 | (0.59,4.28) | 0.356 |  |  |  |  |  |  |  |  |
| Serum Ni | DKD | 8 | 0.940 | (0.52,1.68) | 0.821 |  | 0.780 | (0.42,1.46) | 0.439 |  | 0.290 | (0.08,1.05) | 0.107 |
| Serum Sn^‡^ | DKD | 1 | 4.270 | (0.78,23.4) | 0.093 |  |  |  |  |  |  |  |  |
| Serum Ti | DKD | 5 | 1.080 | (0.70,1.66) | 0.741 |  | 0.860 | (0.49,1.50) | 0.601 |  | 0.560 | (0.19,1.62) | 0.364 |
| Serum Rb | DKD | 4 | 0.850 | (0.51,1.41) | 0.531 |  | 0.890 | (0.49,1.64) | 0.712 |  | 1.090 | (0.34,3.52) | 0.896 |
| Serum Sr | DKD | 8 | 1.030 | (0.57,1.86) | 0.914 |  | 1.040 | (0.58,1.89) | 0.882 |  | 0.440 | (0.08,2.45) | 0.386 |
| Serum Cu | DKD | 6 | 1.210 | (0.77,1.91) | 0.412 |  | 1.140 | (0.63,2.07) | 0.657 |  | 0.660 | (0.09,4.63) | 0.696 |
| Serum Zn | DKD | 5 | 0.890 | (0.53,1.50) | 0.667 |  | 0.990 | (0.51,1.91) | 0.968 |  | 0.280 | (0.00,45.9) | 0.657 |
| Plasma Ba | DKD | 8 | 1.090 | (0.82,1.44) | 0.560 |  | 1.060 | (0.74,1.54) | 0.739 |  | 1.960 | (0.58,6.61) | 0.318 |
| Plasma Pb^†^ | DKD | 2 | 1.160 | (0.73,1.84) | 0.522 |  |  |  |  |  |  |  |  |
| Plasma Co | DKD | 3 | 1.140 | (0.76,1.72) | 0.527 |  | 1.150 | (0.70,1.89) | 0.575 |  | 0.460 | (0.04,5.69) | 0.656 |
| Plasma Mo^†^ | DKD | 2 | 0.900 | (0.54,1.50) | 0.675 |  |  |  |  |  |  |  |  |
| Plasma Cd | DKD | 4 | 1.090 | (0.69,1.72) | 0.703 |  | 0.940 | (0.66,1.35) | 0.746 |  | 1.070 | (0.17,6.59) | 0.945 |
| Plasma V^†^ | DKD | 2 | 0.800 | (0.51,1.26) | 0.335 |  |  |  |  |  |  |  |  |
| Plasma Cr | DKD | 3 | 1.200 | (0.83,1.74) | 0.336 |  | 1.220 | (0.88,1.70) | 0.231 |  | 1.880 | (1.03,3.42) | 0.286 |
| Plasma Al^†^ | DKD | 2 | 0.990 | (0.50,1.96) | 0.981 |  |  |  |  |  |  |  |  |
| Plasma Mn^‡^ | DKD | 1 | 2.290 | (0.94,5.57) | 0.068 |  |  |  |  |  |  |  |  |
| Plasma Ni | DKD | 5 | 0.960 | (0.62,1.51) | 0.872 |  | 0.970 | (0.64,1.49) | 0.902 |  | 2.290 | (0.51,10.3) | 0.360 |
| Plasma Sn^‡^ | DKD | 1 | 2.170 | (0.48,9.76) | 0.312 |  |  |  |  |  |  |  |  |
| Plasma Ti | DKD | 3 | 1.060 | (0.45,2.48) | 0.890 |  | 1.230 | (0.68,2.20) | 0.494 |  | 0.430 | (0.03,5.59) | 0.634 |
| Plasma Rb^†^ | DKD | 2 | 1.560 | (0.76,3.22) | 0.229 |  |  |  |  |  |  |  |  |
| Plasma Sr^†^ | DKD | 2 | 0.770 | (0.44,1.34) | 0.348 |  |  |  |  |  |  |  |  |
| Plasma Cu | DKD | 3 | 0.930 | (0.57,1.52) | 0.767 |  | 0.810 | (0.44,1.48) | 0.490 |  | 2.100 | (0.62,7.14) | 0.445 |
| Plasma Zn | DKD | 3 | 1.390 | (0.00.96,2) | 0.081 |  | 1.350 | (0.88,2.07) | 0.165 |  | 0.990 | (0.14,6.72) | 0.990 |
| DKD | Serum Ba | 4 | -0.001 | 0.004 | 0.766 |  | -0.001 | 0.004 | 0.754 |  | -0.001 | 0.004 | 0.779 |
| DKD | Serum Pb | 4 | 0.000 | 0.004 | 0.874 |  | 0.000 | 0.004 | 0.886 |  | 0.000 | 0.005 | 0.946 |
| DKD | Serum Co | 4 | 0.001 | 0.004 | 0.702 |  | 0.001 | 0.004 | 0.700 |  | 0.000 | 0.005 | 0.877 |
| DKD | Serum Mo | 4 | -0.003 | 0.004 | 0.425 |  | -0.003 | 0.004 | 0.444 |  | -0.004 | 0.004 | 0.428 |
| DKD | Serum Cd | 4 | -0.005 | 0.003 | 0.126 |  | -0.006 | 0.004 | 0.160 |  | -0.003 | 0.004 | 0.425 |
| DKD | Serum V | 4 | -0.005 | 0.004 | 0.179 |  | -0.005 | 0.004 | 0.243 |  | -0.004 | 0.004 | 0.424 |
| DKD | Serum Cr | 4 | 0.000 | 0.004 | 0.888 |  | 0.000 | 0.004 | 0.905 |  | 0.000 | 0.004 | 0.924 |
| DKD | Serum Al | 4 | 0.000 | 0.004 | 0.990 |  | 0.000 | 0.004 | 0.962 |  | -0.002 | 0.004 | 0.605 |
| DKD | Serum Mn | 4 | 0.001 | 0.004 | 0.748 |  | 0.001 | 0.004 | 0.771 |  | 0.002 | 0.004 | 0.631 |
| DKD | Serum Ni | 4 | 0.000 | 0.005 | 0.962 |  | 0.000 | 0.004 | 0.860 |  | -0.001 | 0.006 | 0.789 |
| DKD | Serum Sn | 4 | 0.000 | 0.004 | 0.954 |  | 0.000 | 0.003 | 0.881 |  | 0.002 | 0.003 | 0.628 |
| DKD | Serum Ti | 4 | 0.002 | 0.004 | 0.495 |  | 0.002 | 0.004 | 0.541 |  | 0.002 | 0.004 | 0.616 |
| DKD | Serum Rb | 4 | 0.002 | 0.004 | 0.569 |  | 0.002 | 0.004 | 0.544 |  | 0.002 | 0.006 | 0.679 |
| DKD | Serum Sr | 4 | 0.000 | 0.003 | 0.921 |  | 0.000 | 0.004 | 0.900 |  | 0.000 | 0.004 | 0.873 |
| DKD | Serum Cu | 4 | 0.001 | 0.004 | 0.626 |  | 0.001 | 0.004 | 0.647 |  | 0.001 | 0.004 | 0.719 |
| DKD | Serum Zn | 4 | 0.000 | 0.004 | 0.920 |  | 0.000 | 0.004 | 0.906 |  | 0.000 | 0.004 | 0.995 |
| DKD | Plasma Ba | 3 | -0.033 | 0.112 | 0.768 |  | 0.011 | 0.090 | 0.896 |  | -0.488 | 0.204 | 0.252 |
| DKD | Plasma Pb | 3 | -0.041 | 0.100 | 0.677 |  | -0.040 | 0.084 | 0.632 |  | -0.412 | 0.192 | 0.277 |
| DKD | Plasma Co | 3 | -0.055 | 0.067 | 0.405 |  | -0.067 | 0.074 | 0.364 |  | -0.286 | 0.196 | 0.382 |
| DKD | Plasma Mo | 3 | -0.029 | 0.119 | 0.801 |  | 0.009 | 0.089 | 0.915 |  | 0.005 | 0.516 | 0.992 |
| DKD | Plasma Cd | 3 | -0.003 | 0.073 | 0.965 |  | -0.035 | 0.087 | 0.688 |  | -0.062 | 0.310 | 0.873 |
| DKD | Plasma V | 3 | 0.063 | 0.067 | 0.348 |  | 0.070 | 0.077 | 0.362 |  | 0.211 | 0.207 | 0.492 |
| DKD | Plasma Cr | 3 | -0.025 | 0.067 | 0.707 |  | -0.028 | 0.078 | 0.715 |  | -0.022 | 0.206 | 0.931 |
| DKD | Plasma Al | 3 | 0.023 | 0.088 | 0.787 |  | -0.055 | 0.086 | 0.519 |  | -0.218 | 0.285 | 0.583 |
| DKD | Plasma Mn | 3 | -0.042 | 0.067 | 0.526 |  | -0.051 | 0.082 | 0.534 |  | -0.256 | 0.205 | 0.429 |
| DKD | Plasma Ni | 3 | -0.039 | 0.104 | 0.703 |  | 0.022 | 0.084 | 0.789 |  | -0.468 | 0.202 | 0.259 |
| DKD | Plasma Sn | 3 | -0.019 | 0.111 | 0.858 |  | -0.032 | 0.082 | 0.693 |  | 0.416 | 0.207 | 0.294 |
| DKD | Plasma Ti | 3 | 0.085 | 0.110 | 0.437 |  | 0.060 | 0.093 | 0.517 |  | 0.509 | 0.206 | 0.244 |
| DKD | Plasma Rb | 3 | -0.067 | 0.110 | 0.543 |  | -0.049 | 0.083 | 0.554 |  | -0.498 | 0.185 | 0.226 |
| DKD | Plasma Sr | 3 | -0.077 | 0.066 | 0.245 |  | -0.065 | 0.077 | 0.396 |  | 0.103 | 0.205 | 0.703 |
| DKD | Plasma Cu | 3 | -0.015 | 0.081 | 0.852 |  | -0.031 | 0.080 | 0.697 |  | -0.123 | 0.336 | 0.775 |
| DKD | Plasma Zn | 3 | -0.024 | 0.067 | 0.718 |  | -0.026 | 0.078 | 0.738 |  | -0.227 | 0.206 | 0.469 |
| Blood Al | DKD | 4 | 1.040 | (0.92,1.18) | 0.543 |  | 1.090 | (0.92,1.30) | 0.299 |  | 1.000 | (0.78,1.28) | 0.995 |
| Blood Cd | DKD | 7 | 1.094 | (0.97,1.23) | 0.134 |  | 1.160 | (1.03,1.32) | **0.018*** |  | 1.090 | (0.86,1.38) | 0.512 |
| Blood Co | DKD | 3 | 0.932 | (0.77,1.13) | 0.473 |  | 0.950 | (0.74,1.21) | 0.665 |  | 0.910 | (0.52,1.60) | 0.806 |
| Blood Cr | DKD | 3 | 0.938 | (0.84,1.05) | 0.272 |  | 0.930 | (0.82,1.06) | 0.287 |  | 0.960 | (0.57,1.62) | 0.903 |
| Blood Cu | DKD | 9 | 0.989 | (0.93,1.05) | 0.736 |  | 0.980 | (0.92,1.05) | 0.644 |  | 0.940 | (0.86,1.03) | 0.207 |
| Blood Hg | DKD | 3 | 1.025 | (0.93,1.13) | 0.620 |  | 1.030 | (0.94,1.13) | 0.543 |  | 1.130 | (0.88,1.44) | 0.522 |
| Blood Mn | DKD | 6 | 1.126 | (1.00,1.27) | 0.056 |  | 1.170 | (1.00,1.36) | **0.044*** |  | 1.200 | (0.92,1.58) | 0.249 |
| Blood Mo | DKD | 4 | 0.937 | (0.83,1.06) | 0.287 |  | 0.940 | (0.82,1.09) | 0.416 |  | 0.930 | (0.75,1.16) | 0.607 |
| Blood Ni | DKD | 8 | 0.992 | (0.94,1.05) | 0.759 |  | 0.980 | (0.93,1.04) | 0.492 |  | 0.930 | (0.84,1.03) | 0.215 |
| Blood Pb | DKD | 3 | 0.923 | (0.67,1.27) | 0.615 |  | 1.080 | (0.80,1.46) | 0.628 |  | 1.070 | (0.41,2.79) | 0.909 |
| Blood Zn | DKD | 4 | 0.992 | (0.80,1.23) | 0.935 |  | 0.930 | (0.78,1.11) | 0.427 |  | 0.650 | (0.46,0.92) | 0.139 |
| nSNPs, number of single nucleotide polymorphisms; IVW, inverse variance weighting; OR, Odds Ratio; CI, Confidence Interval; SE, standard error; DKD, Diabetic Kidney Disease.  When nSNPs = 2, only the IVW method is used for Mendelian randomization analysis; when nSNPs = 1, only the Wald ratio method is used for Mendelian randomization analysis.  ^†^ Only the IVW method is used for Mendelian randomization analysis  ^‡^ Only the Wald ratio method is used for Mendelian randomization analysis.  ^1^When the outcome is DKD, use OR and 95% CI to describe the results.  ^2^When the outcome is heavy metal, use Beta and SE to describe the results.  *P*-value, the significance level of mendelian randomization analysis results.  **P*-value < 0.05. | | | | | | | | | | | | | |

# Supplementary Figures

**
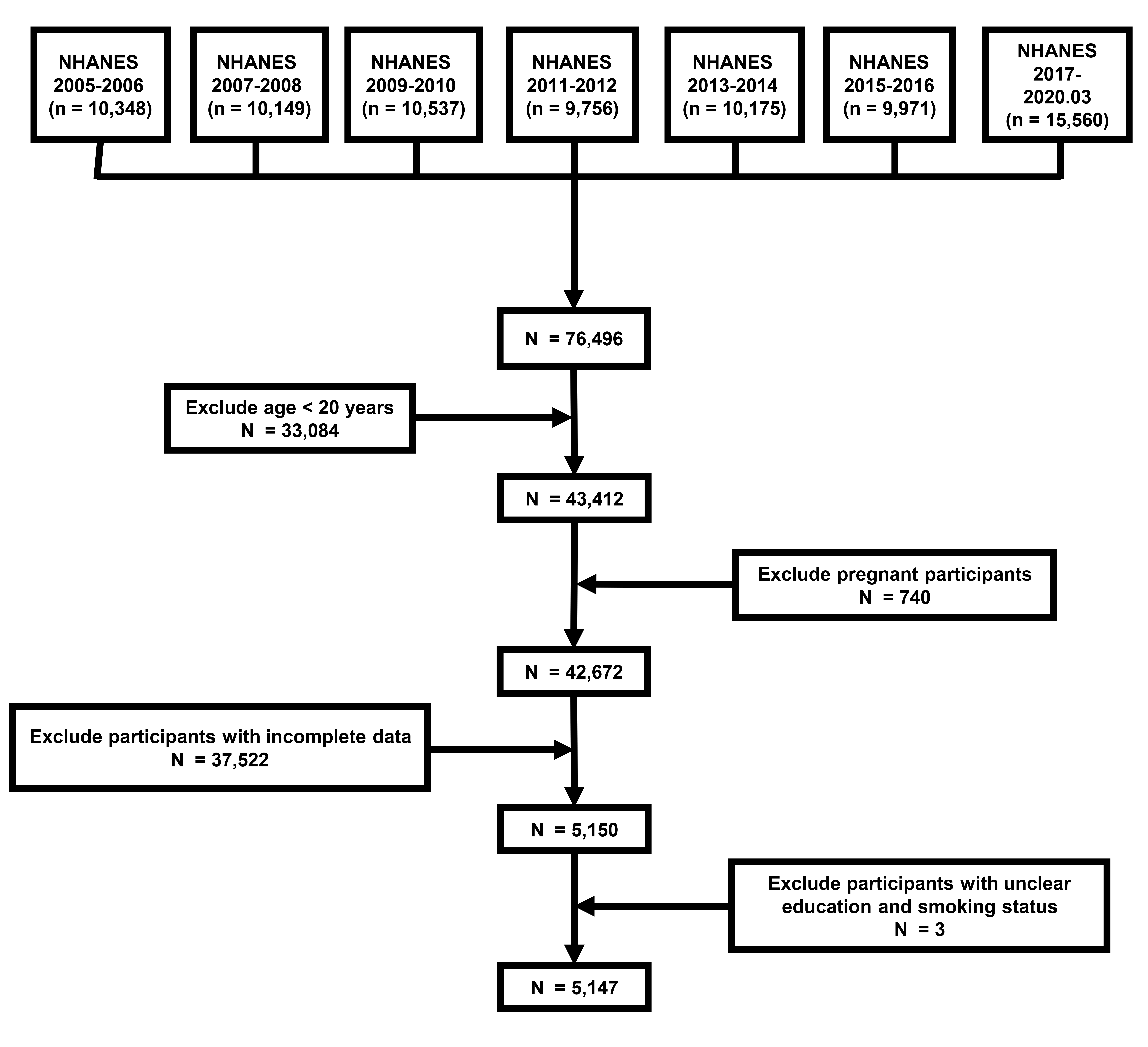
Figure S1.** Flow chart of selection process for NHANES 2005.01-2020.03.

**
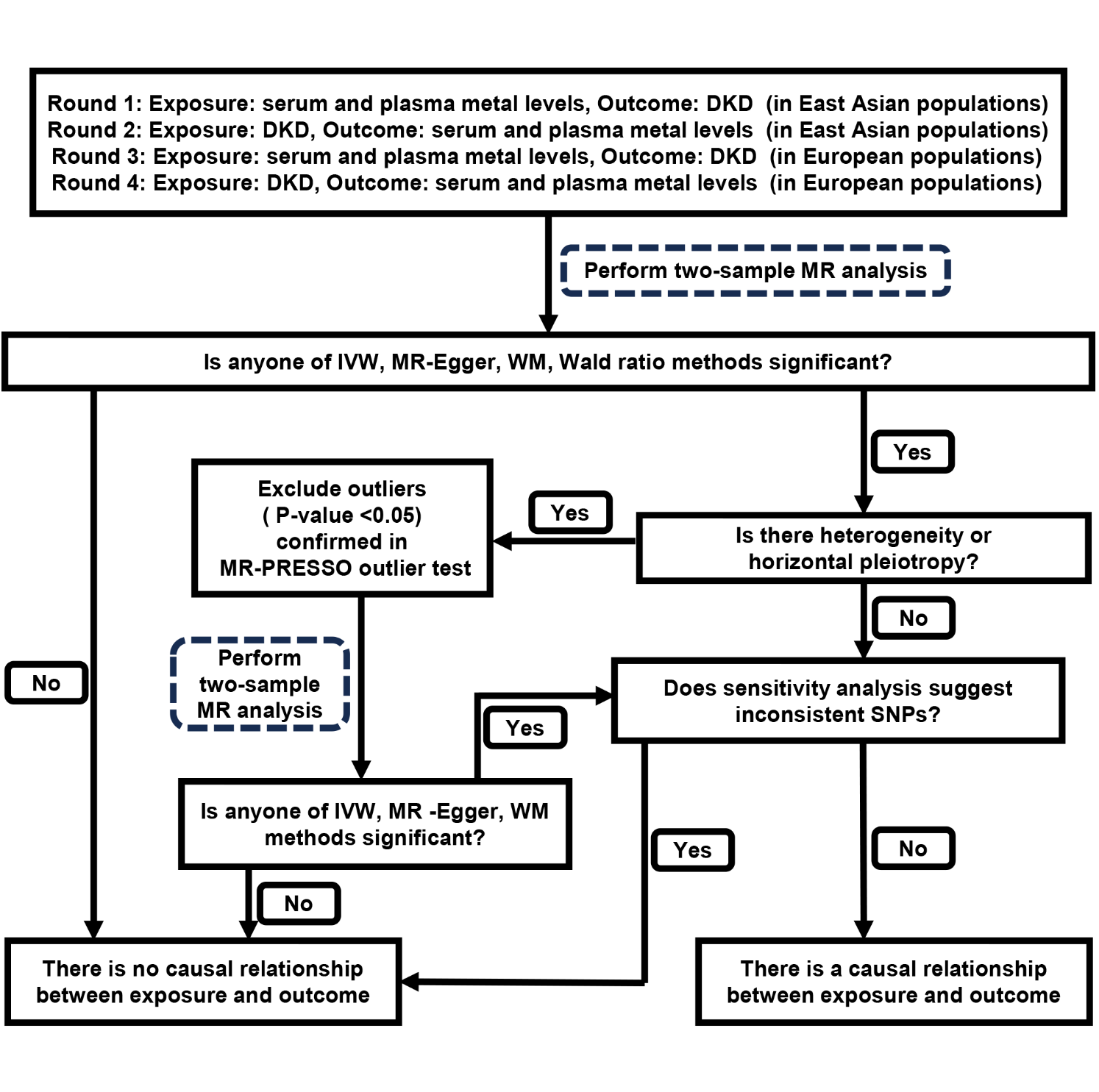
****Figure S2.** Flow chart of the two-sample Mendelian randomization analysis.

**
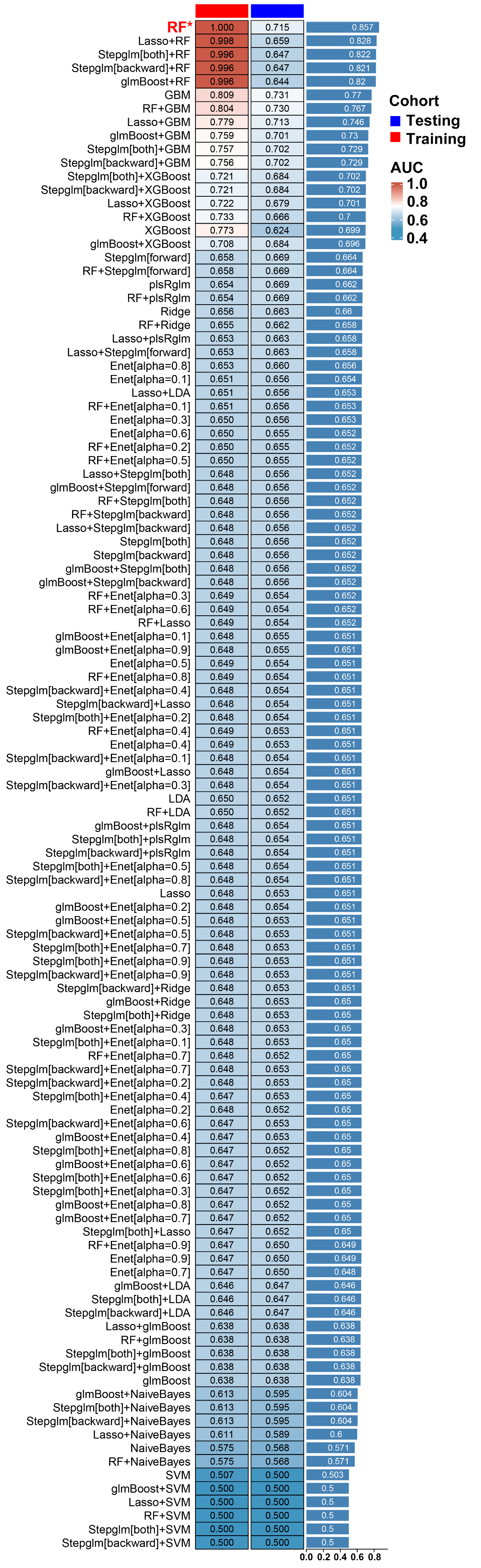
F****igure S3.**

Heatmap of AUC values of machine learning

prediction models for 9 urinary heavy metals exposures.
